# *In vitro* immune evaluation of adenoviral vector-based platform for infectious diseases

**DOI:** 10.5114/bta.2023.132775

**Published:** 2023-12-21

**Authors:** Joanna Baran, Łukasz Kuryk, Teresa Szczepińska, Michał Łaźniewski, Mariangela Garofalo, Anna Mazurkiewicz-Pisarek, Diana Mikiewicz, Alina Mazurkiewicz, Maciej Trzaskowski, Magdalena Wieczorek, Katarzyna Pancer, Ewelina Hallmann, Lidia Brydak, Dariusz Plewczynski, Tomasz Ciach, Jolanta Mierzejewska, Monika Staniszewska

**Affiliations:** 1 Centre for Advanced Materials and Technologies, Warsaw University of Technology, Warsaw, Poland; 2 National Institute of Public Health, Warsaw, Poland; 3 University of Padova, Padova, Italy; 4 Faculty of Chemical and Process Engineering, Warsaw University of Technology, Warsaw, Poland; 5 Faculty of Mathematics and Information Science, Warsaw University of Technology, Warsaw, Poland; 6 Centre of New Technologies, University of Warsaw, Warsaw, Poland; 7 Faculty of Chemistry, Warsaw University of Technology, Warsaw, Poland

**Keywords:** adenoviral vectors, vaccine platform, innate and adaptive immunity

## Abstract

New prophylactic vaccine platforms are imperative to combat respiratory infections. The efficacy of T and B memory cell-mediated protection, generated through the adenoviral vector, was tested to assess the effectiveness of the new adenoviral-based platforms for infectious diseases. A combination of adenovirus AdV1 (adjuvant), armed with costimulatory ligands (ICOSL and CD40L), and rRBD (antigen: recombinant nonglycosylated spike protein rRBD) was used to promote the differentiation of T and B lymphocytes. Adenovirus AdV2 (adjuvant), without ligands, in combination with rRBD, served as a control. *In vitro* T-cell responses to the AdV1+rRBD combination revealed that CD8+ platform-specific T-cells increased (37.2 ± 0.7% vs. 23.1 ± 2.1%), and T-cells acted against SARS-CoV-2 via CD8+TEMRA (50.0 ± 1.3% vs. 36.0 ± 3.2%). Memory B cells were induced after treatment with either AdV1+rRBD (84.1 ± 0.8% vs. 82.3 ± 0.4%) or rRBD (94.6 ± 0.3% vs. 82.3 ± 0.4%). Class-switching from IgM and IgD to isotype IgG following induction with rRBD+Ab was observed. RNA-seq profiling identified gene expression patterns related to T helper cell differentiation that protect against pathogens. The analysis determined signaling pathways controlling the induction of protective immunity, including the MAPK cascade, adipocytokine, cAMP, TNF, and Toll-like receptor signaling pathway. The AdV1+rRBD formulation induced IL-6, IL-8, and TNF. RNA-seq of the VERO E6 cell line showed differences in the apoptosis gene expression stimulated with the platforms vs. mock. In conclusion, AdV1+rRBD effectively generates T and B memory cell-mediated protection, presenting promising results in producing CD8+ platform-specific T cells and isotype-switched IgG memory B cells. The platform induces protective immunity by controlling the Th1, Th2, and Th17 cell differentiation gene expression patterns. Further studies are required to confirm its effectiveness.

## Introduction

Viruses possess one or more properties that enable them to diminish the efficiency of host adaptive or innate immunity, and we lack effective vaccines against most of these agents (Rouse and Sehrawat, 2010). Throughout vaccine development, the immunogenicity of the vaccine candidate is monitored via *in vitro* studies, animal studies, and clinical trials (Tapia-Calle et al., 2017). Given that vaccines sometimes fail in clinical trials despite success in animal experiments, it is imperative to study *ex vivo* human-derived cell responses in both qualitative and quantitative characteristics (Watkins et al., 2008). Rational vaccine design against pandemics and emerging infections demands insight into the mechanisms by which vaccines and adjuvants are sensed by the innate immune system and how they stimulate adaptive immunity (Pulendran and Ahmed, 2011). Clinical studies have revealed differences in T-cell and antibody responses in sera from individuals vaccinated with a single/double dose of ChAdOx1 nCoV-19 (Oxford-AstraZeneca) and BNT16262 (Pfizer-BioNTech) (Ewer et al., 2021; Saleem et al., 2022). Studies indicated that Ad26.COV2.S (Johnson and Johnson) and mRNA-1273 (Moderna) induced the CD4^+^ and CD8^+^ T-cell response (Fiolet et al., 2022). Moreover, Sputnik Light (Gamaleya Institute) induced strong humoral and cellular immune responses in both seropositive and seronegative participants (Logunov et al., 2020). According to the WHO, the CanSinoBio Ad5 nCoV-S vaccine (Convidencia^TM^) induced a cellular response in at least 91% of study participants (WHO, 2022). The manner in which adenovirus vectors mediate their immunogenicity remains unidentified (Pulendran and Ahmed, 2011). Thus, our research identified the role of the innate immune system in sensing platform factors: the antigen (recombinant nonglycosylated spike protein rRBD) and adjuvants (adenoviruses), and in programming protective immune responses. Ke et al. (2022) demonstrated that the recombinant nonglycoRBD protein provided a robust immune response and elicited neutralizing antibodies.

Adenovirus vectors are particularly interesting for their potential applications in human gene therapy (Afkhami et al., 2016; Siggins et al., 2021). We previously designed adenovirus AdV-D24-inducible costimulator ligand (ICOSL)-CD40L (AdV1), which selectively replicated in cancer cells but not in healthy cells and was armed with two potent costimulatory molecules: inducible costimulator ligand (ICOSL) and CD40 ligand (CD40L, CD154) (Garofalo et al., 2021). The inducible costimulator (ICOS) is a CD28-related molecule expressed on activated T cells and is capable of interacting with its ligand ICOSL, present on APCs such as like dendritic cells (DCs), B lymphocytes, and certain cancer cells (Huang et al., 2019). Additionally, the interaction of CD40, expressed on B cells, macrophages, and DCs, with CD40L leads to the activation of adaptive immune responses, including the development of CD8^+^ cytotoxic T lymphocytes (CTLs) (Mohib et al., 2020).

We explored several mechanisms through which adenoviruses (AdV1 or AdV2 without ICOSL and CD40L) function as adjuvants. We conducted *ex vivo* studies to evaluate the efficiency of AdVs when combined with the recombinant spike protein rRBD, focusing on the induction of innate and adaptive immunity. Using a flow cytometry-based assay, we assessed the percentages of CD4^+^, CD8^+^, and CD19^+^ subpopulations. Specifically, the study evaluated the capacity for platform-specific T-cell production. While T memory cells provide protection against subsequent viral infections (Shane and Klonowski, 2014) and modify antibody targets, the efficiency of T memory cells, when primed by adjuvants, was examined.

Additionally, we investigated whether AdVs (adjuvants) in combination with the recombinant spike protein rRBD utilize one or more of the following mechanisms to invoke an immune response: 1) CD8^+^ and CD4^+^T cell activation and differentiation into respective populations, 2) development and differentiation of B cells, and 3) cell apoptosis, which plays a crucial role in controlling immunity by presenting antigens to T cells effectively. The central issue was whether adjuvants stimulate the immune response necessary for protection, such as cytotoxic T cells, long-term memory T cells, or B cells. It was important to understand whether stimulating the peripheral blood mononuclear cell (PBMC) with the platforms (immunogenic factors) affects T cells and if it could confer enduring effects on the antiviral capacity of the CD4^+^ and CD8^+^ T cells. Considering that the study of T cells in preventing COVID-19 is still nascent, we characterized the central memory T_CM_, effector memory T_EM_, and effector memory cells re-expressing CD45RA T_EMRA,_ which were targeted by the adjuvants. We sought to deepen our understanding of T-cell differentiation and to discern how to selectively manipulate this pool for vaccine development. Among several mechanisms proposed to underpin vaccine development, the most persuasive evidence indicated the role of inflammatory cytokines in reducing the risk of vaccinepreventable diseases and their sequelae. Therefore, we studied the immunologic mechanism behind vaccinations, including the production of a series of cytokines influencing the effector cytotoxic T cells or B cells. Moreover, to understand the mode of action of the proposed adjuvants in immunity, we employed RNA-Seq for the transcript quantification in the VERO E6 cell line and utilized RT-qPCR to measure the CD40 transcript at various times during treatment with the platform factors.

## Material and methods

### Viruses, media, and rRBD. Vaccine platform design

The VERO E6 ATCC cells, sourced from LGC Standard (Lomianki, Poland), were utilized as an infection model (Ogando et al., 2020). These cells were cultured in Eagle’s Minimum Essential Media (EMEM) ATCC (LGC Standard, Lomianki, Poland), enriched with 1% penicillin/streptomycin (Gibco Laboratories, USA) and 10% fetal bovine serum (FBS, Gibco Laboratories, USA). Two adenoviruses were employed at stock con-centrations: AdV-D24-ICOSL-CD40L, designated AdV1 (5.2 × 10^11^ VP/ml), and AdV5.3-d24-E3, designated AdV2 (7.7 × 10^12^ VP/ml) (Garofalo et al., 2021). Visualization of the viruses was accomplished using scanning electron microscopy with an SEM Hitachi SU8230 (Japan) and is documented in supplementary Figure S1. AdV1 and Adv2 particle counts were determined through UV spectrophotometry (Tecan, Männedorf, Switzerland) following the method described by Sobotka et al. (2022), utilizing an extinction coefficient of 1.1 × 10^12^ viral particles per OD 260 unit. We calculated VP using Equation: VP = A260 × dilution factor × 1.1 × 10^12^/ml, where the 260/280 nm ratio equaled 2.0, and the absorbance at 260 nm was between 0.1 and 1.0 OD unit. Characterization of adenoviruses involved titration (VP/ml) in the NCI-H226 cell line, cultivated in RPMI 1640 Medium (Gibco Laboratories, USA), supplemented with 1% penicillin/streptomycin (Gibco Laboratories, USA) and 10% FBS. The OD-260-SDS method was used to determine the concentration of virus particles (VP/ml) in NCI-H226 cells (extinction coefficient of 1.1 × 10^12^/Abs 260 unit) grown in ATCC-formulated RPMI-1640 Medium (ATCC, USA), supplemented with FBS (ATCC, USA) to a final concentration of 10% (Sweeney and Hennessey, 2002; Garofalo et al., 2021). Additionally, the TCID50 assay was conducted to quantify viral titers by determining the concentration causing a cytopathic effect (CPE) in 50% of infected cells: AdV1 displayed 3.2 × 10^3^ TCID50/ml and AdV2 exhibited 3.2 × 10^6^ TCID50/ml (supplementary Figs. S2, S3 and Table S1). Given the high variability observed in these assays, a ratio of TCID50/VP = 1 : 9 was adopted, and the study ratio VP/ml/IU/ml was used and found to be comparable between the tested stocks.

Adenoviruses’ purification: Infected cell pellets were resuspended in 10 mM Tris, pH 8.0, and subjected to three freeze/thaw cycles to release virus particles. The infected cell lysate was then loaded onto a two-step CsCl gradient in an SW28 Beckman tube. Following a 2-h centrifugation at 20 000 rpm, the virus band was collected and loaded onto a continuous CsCl gradient in an SW41 Beckman tube. After overnight centrifugation at 20 000 rpm, the band obtained from the second gradient was immediately dialyzed against 4 × 0.5 l GTS buffer (2.5% glycerol, 25 mM NaCl, 20 mM Tris-HCl, pH 8.0) for ~18 h at 4°C. About 1.3 ml of the dialyzed virus suspension was collected, filtered through a 0.22 μm Supor membrane (Pall, MI), and the virus was frozen at —70°C.

### Expression of rRBD gene in the E. coli strain

Genetic engineering methods were utilized to construct the recombinant Receptor Binding Domain (rRBD) gene in the prokaryotic expression vector. The gene sequence, designed for protein expression, was based on the amino acid sequence. According to literature data (Yuan et al., 2020), the region of the SARS-CoV-2 virus coding protein with the highest probability of inducing an immune response was selected (amino acids 331–524 of the SARS-CoV-2 S protein, GenBank: QHD43416.1). The T4 folded protein (F4) was added to increase the probability of correct assembly of the protein’s tertiary structure. The tag at the N-terminus allowed the use of a simple affinity chromatography method to purify the proteins. Restriction sites (NdeI, XbaI) were added. The nucleotide sequence of the genes was optimized for bacterial codon usage and inserted into the pUC57 vector (GeneScript, Rijswijk, Netherlands). Vector pUC57, with the RBD+F4+6histag encoding sequence, was transformed into *E. coli* DH5α competent cells and isolated using standard techniques (Sambrook et al., 1989).

Vector pUC57 was digested with NdeI/XbaI restriction enzymes (New England Biolabs, UK Ltd.) at 37°C according to the manufacturer’s instructions and then applied to a 1% agarose gel. According to the manufacturer’s instructions, the digested DNA fragment of 696 bp was isolated using the Gel-Out Kit (A&A Biotechnology, Poland). Vector pDM, digested with NdeI/XbaI, was utilized for ligation (T4 DNA ligase; Roche, Swiss) with the DNA fragment digested with the restriction enzymes NdeI/XbaI, containing the sequence of the rRBD gene (supplementary Table S2). The ligation mixture was transformed into *E. coli* NEB Turbo (New England Biolabs, UK Ltd.) competent cells, following the manufacturer’s instructions. Plasmid DNA was isolated from the bacterial colonies using a Plasmid Mini Isolation Kit (A&A Biotechnology, Poland) in alignment with the manufacturer’s instructions. The accuracy of DNA sequences was confirmed by sequence analysis (Genomed, Poland). The pDM/RBD expression vector was transformed by electroporation into *E. coli* competent cells. Breeding conditions were as follows: 37°C for 1 h, 10 g, LB medium, supplemented with tetracycline (100 μg/ml). Plates were incubated at 37°C overnight. A single colony was selected and cultured in 50 ml of LB medium with tetracycline added to prepare stocks. The breeding was conducted at 37°C with shaking until the optical density (OD = 600 nm) reached about 0.6–0.8, and then suspended in 50% glycerol in a 1 : 1 ratio. The stocks were stored at —70°C.

After optimizing the culture under various conditions, the selected *E. coli* expression strain breeding conditions were 30°C, 18 h, and LB medium (data not published). The bacterial media utilized for research were as follows: LB agar medium (10 g/l bacto tryptone; 5 g/l yeast extract; 10 g/l NaCl; BD USA) + 1.5 g/100 ml agar, and liquid LB medium (10 g/l bacto tryptone; 10 g/l yeast extract; 5 g/l NaCl, BD USA; 100 g/l PEG 6000; pH 6.1; Merck, Germany). Antibiotics used for research included ampicillin (Amp; Merck, Germany) at 100 μg/ml, and tetracycline (Tet, Merck, Germany) at 100 μg/ml. The recombinant RBD protein was obtained in the form of inclusion bodies. A method for isolating inclusion bodies and purifying recombinant protein using Ni-NTA affinity chromatography was developed. Cells were harvested by centrifugation at 15 000 × g for 15 min at 4°C. The pelleted cells were suspended in lysis buffer (50 mM Tris-HCl pH 8.0, 500 mM NaCl, 1 mM EDTA, 0.043% lysozyme, 1% PMSF protease inhibitor; Merck, Germany). The bacterial suspension was gently mixed for 30 min at room temperature. Then, 20 ml of Triton X-100 (Merck, Germany) was added, and the suspension was stirred for 10 min. Cells were lysed by a high-pressure homogenizer and centrifuged at 18 400 × g for 15 min at 4°C. The pellets were washed with 50 mM Tris-HCl pH 8.0, 500 mM NaCl, and 1% Triton X-100 (Merck, Germany), and then centrifuged again at 18 400 × g for 15 min at 4°C. Subsequently, the pellets were washed twice with 50 mM Tris-HCl pH 8.0, 500 mM NaCl buffer (Merck, Germany). Finally, the inclusion body suspension was centrifuged at 15 000 × g for 15 min at 4°C. The obtained inclusion bodies were frozen at —20°C for further preparation.

The inclusion bodies containing rRBD protein were dissolved in 50 mM phosphate buffer pH-12 with 7 M urea and 5 mM β-mercaptoethanol (Merck, Germany) and stirred for 45 min at room temperature. The pH of the solution was adjusted to 8.0 with 5 M HCl (Merck, Germany). Lastly, the suspension was centrifuged at 24 000 × g for 15 min at 4°C to remove insoluble debris. Recombinant RBD was purified by NiNTA Sepharose (Qiagen, Germany) chromatography. The elution buffer comprised 50 mM phosphate buffer, 7 M urea, 300 mM NaCl, and 300 mM imidazole (Merck, Germany). After purification, rRBD was left to fold in a dialyzing buffer (50 mM phosphate buffer pH 8.0, 10% glycerol; Merck, Germany) for 24 h with stirring. The overexpression of the rRBD gene was confirmed by separating cellular proteins using 15% SDS-PAGE and by mass spectrometry (supplementary Fig. S4).

### Human sample collection and processing

Studies utilized commercially available buffy coat pooling sets to prepare peripheral blood mononuclear cells (PBMC). Buffy coats, derived from the whole blood of healthy donors, were purchased from the Regional Centre for Blood and Blood Treatment in Warsaw, Poland. In brief, PBMCs were isolated from fresh heparinized blood through Ficoll-Hypaque density gradient centrifugation and subsequently frozen. The cells were cryopreserved in a freezing medium, composed of 50% (vol/vol) fetal bovine serum (Invitrogen, USA) in OptiMEM medium (Invitrogen, Waltham, MA, USA), and 20% DMSO, then stored at —140°C until further analysis. For analysis, frozen aliquots of PBMC were incubated for 1 min in a 37°C water bath. The thawed PBMCs were rested as previously described (Tapia-Calle et al., 2017), with cell viability exceeding 80% in all samples. The cells were then seeded at a concentration of 1 × 10^6^/ml in 24-well plates in Opti-MEM medium (Invitrogen, Waltham, MA, USA), containing 50% (vol/vol) FBS (Invitrogen, USA) and 20% DMSO, and incubated for 24 h at 37°C and 5% CO_2_. After a 24-h resting period, PBMCs underwent separate treatments with the following: 1) mock stimulation with LPS (1.25 μg/ml); 2) Ad5/3-D24-ICOS-CD40L (AdV1) at 100 VP/ml (stock 3.2×10^3^ TCID_50_/ml); 3) Ad-D24-WT (AdV2) at 100 VP/ml (3.2 × 10^6^ TCID_50_/ml); 4) rRBD at 2.62 μg/ml without neutralizing Ab (2.62 μg/ml); 5) AdV1 (100 VP/ml) and rRBD (IC_50_ = 2.62 μg/ml); 6) AdV2 (100 VP/ml) and rRBD (IC50 = 2.62 μg/ml). Each component, in the presence of LPS (1.25 μg/ml), was tested and incubation was performed for either 24 h or 7 days at 37°C and 5% CO_2_.

### Flow cytometry FACS Lyric flow cytometer

Flow cytometry was performed using a BD Lyric FACS Flow (BD Bioscience, NJ, USA). PBMCs were washed with BD^®^ CellWASH buffer (BD Biosciences) and stained with a cocktail of surface antibodies, adhering to the modified *Protocols for Multicolor Immunofluorescent Staining of Cells Using BD Horizon Brilliant Stain Buffer Plus* (BD Biosciences, cat. no. 566385). In brief, 50 μl of PBMCs (1 × 10^6^ cells/ml) were incubated at room temperature in the dark for 30 min with the following antibodies: CD3 (APC-H7, cat. no. 560176), CD8 (PerCP, cat. no. 345774), CD4 (PE-Cy7, cat. no. 557852), CD197 (BB515, cat. no. 566764), CD45RA (APC, cat. no. 550855), and CD95 (PE, cat. no. 555674) (all from BD Biosciences USA). Subsequently, cells were washed using BD^®^ CellWASH buffer (BD Biosciences). In every instance, 100,000 gated events were acquired and data were analyzed using FACSLyric software. Subsets of memory CD4^+^ and CD8^+^ T cells in PBMCs were identified based on CCR7 (CD197) and CD45RA, allowing the discrimination of CD45RA^-^CD197^+^, central memory T cells (T ); CD45RA^-^CD197^-^, effector memory T cells; CD45RA^+^CD197^-^,terminally differentiated effector memory T cells (T_EMRA_); CD45RA^+^CD197^+^ CD95^+^, memory stem cells (T_SCM_); and CD45RA^+^CD197^+^ CD95^-^, NAÏVE cells.

After vaccination or infection, a search was conducted for antigen-specific B cells, such as plasmablasts. They were identified by surface staining as CD19^+^CD20^-^ CD27^+^CD38^+^. Approximately 10^6^ cells were resuspended in 100 μl of staining buffer BD, containing Brilliant™ Stain Buffer (BD Biosciences, cat. no. 563794), Stain Buffer (FBS, BD Biosciences), and the following surface antibodies for panel 2, in the volumes recommended by the producer: CD24^+^ (BB515, cat. no. 564521), CD38^+^ (PE, cat. no. 555460), CD19^+^ (BB700, cat. no. 566396), IgD^+^ (PE-Cy7, cat. no. 561314), CD27^+^ (APC, cat. no. 558664), CD20^+^ (APC-Cy7, cat. no. 335829) and for panel 3: IgM (BB515, cat. no. 564622), CD38^+^ (PE, cat. no. 555460), CD19^+^ (BB700, cat. no. 566396), IgD^+^ (PE-Cy7, cat. no. 561314), CD27^+^ (APC, cat. no. 558664), and IgG^+^ (APC-H7, cat. no. 561297). The incubation was carried out for 20 min, protected from light.

The BD Cytometric Bead Array (CBA) Human Soluble Protein Master Buffer Kit (BD Biosciences, cat. no. 558264) was used to capture a set of soluble analytes/cytokines utilizing beads of known size and fluorescence via flow cytometry using the FACS Lyric BD (Shin et al., 2007). Briefly, the array incorporated tests for proinflammatory cytokines (IL-6, IL-8, IFN(, TNF, IL-1α, IL-1β, and IL-12 p70) and anti-inflammatory cytokines (IL-2, IL-4, and IL-10). The BD CBA standards were reconstituted and serially diluted, followed by mixing with Capture Beads and the Detection Reagent. The Capture Beads BD CBA Human Soluble Protein Flex Set (50 × conc.) were diluted in accordance with the producer’s protocol (BD Biosciences, cat. no. 558264) and mixed before being transferred to test tubes as directed by the manufacturer (Table 1).

**Table 1:** Protein concentration in standard tubes

Dilution	Standard tubes									
	top standard	1 : 2	1 : 4	1 : 8	1 : 16	1 : 32	1 : 64	1 : 128	1 : 256	0
Protein concentration [pg/ml]	2.500	1.250	625	312.5	156	80	40	20	10	0

Detection reagents and human soluble protein flex sets were prepared following the provided instructions (BD Biosciences, cat. no. 558264). Data were analyzed using FCAP Array v 3 software (BD Biosciences). Levels of cytokines within the same mixture were determined simultaneously, as beads that detect a specific cytokine exhibit distinct fluorescence intensity. The amount of each cytokine in the supernatant was interpolated from a standard curve (supplementary Fig. S5A–M), generated with each recombinant cytokine, using the FCAP Array software (BD Biosciences, NJ, USA).

### Reverse transcription quantitative real-time polymerase chain reaction (RT-qPCR) and RNA sequencing (RNA-seq)

RT-qPCR: RNA was extracted from fresh VERO E6 cell pellets using the Total RNA Mini Kit (A&A Biotechnology, Gdansk, Poland) and reverse-transcribed using the High-Capacity cDNA Reverse Transcription Kit with RNase Inhibitor (ThermoFisher Scientific, Waltham, USA). cDNA was diluted to achieve similar levels of the GAPDH reference gene, and subsequently, 1 μl was added to 20-μl PCR reactions containing random primers, MultiScribe™ Reverse Transcriptase, and buffer (ThermoFisher Scientific, Waltham, USA, Cat. 4374966). Real-time PCR was performed on a CFX96 (Bio-Rad, USA) with an initial step of 5 min at 95°C, followed by 45 cycles of: 95°C for 30 s, 61°C for 1 min, 72°C for 1 min, and a melting curve from 50 to 95°C. The GAPDH reference gene was run alongside each target to normalize target gene levels relative to mRNA levels (Table 2).

**Table 2:** Primer sequences

Gene		Primer sequence
GAPDH	forward	5’–TGGACTCCACGACGTACTCA–3’
	reverse	5’–ATGCTGCATTCGCCCTCTT–3’
CD40	forward	5’–GAGGCTGCAAATGGAAGTGC–3’
	reverse	5’–GCTGCTGGAGTCCCCATATC–3’

RNA-Seq: We prepared 1 × 10^6^ cryo-conserved VERO E6 cells, treated with platform factors (plus an untreated control), for RNA isolation, obtaining 25 μl of total volume per sample, each containing ≥1 μg RNA with a concentration of ≥ 40 ng/μl. Sequencing was conducted on the NovaSeq 6000; read length: 2 × 100 bp, output: 50 M clusters (10 Gb) per sample. Raw data were delivered as trimmed FASTQ files (CeGaT GmbH, Tübingen, Germany). For RNA-seq analysis, reads were trimmed from both ends if their quality in the Phred scale was below 30; subsequently, only reads longer than 40 bp were retained (cutadapt 3.5-m 40–qualitybase–30). Reads were aligned to the VERO_WHO_p1.0 reference genome using the align function from the Rsubread package (ver. 2.8.2) (Liao et al., 2019). Only reads with flags 99, 147, 83, or 163 were retained for further analysis. Reads were assigned to genes, as defined by the RefSeq annotation (GCF_015252025.1_VERO_WHO_p1.0_genomic.gtf), using the featureCounts function. Differential expression was performed using the DESeq2 (ver. 1.34.0) R package (Love et al., 2014). Initially, samples were grouped into three categories to assess common dispersion in the experiment since each condition had only one replicate. Group 1: VERO E6 cell line untreated (control); Group 2: AdV1 IC100, Pseudo-SARS-CoV-2 IC100, AdV1+pseudo-SARS-CoV-2 IC100; Group 3: AdV2 IC100, AdV1+pseudoSARS-CoV-2 IC100. Common dispersion was estimated to be 0.002349 and was used to calculate differential expression between each condition and control using the exact test. Unexpressed genes were filtered with the filterByExpr function, leaving 18 914 genes in the analysis. False Discovery Rate values were adjusted using the Benjamini–Hochberg method. For each group, gene ontology enrichment in relation to control was analyzed separately with GPrifiler (https://biit.cs.ut.ee/gprofiler/gost, g:Profiler version e106_eg53_p16_65fcd97, database updated on 18/05/2022, with *Chlorocebus sabaeus* (Vervet AGM) gene background, (Raudvere et al., 2019) for the gene group demonstrating significantly changing expression in each condition separately.

### Statistical analysis

Statistical analysis was conducted on both quantitative and qualitative data. Unless otherwise specified, continuous data are expressed as the mean ± SD. Multiple tests to evaluate various variables were executed, including the multiple unpaired *t*-test, Wilcoxon signed-rank test, paired *t*-test, RM one-way ANOVA, and correlation (Pearson r). These analyses were performed to identify any significant variations in variables between groups (e.g., mock stimulated with LPS vs. marked treatment). All statistical tests yielding *P*-values ≤0.05 were considered significant. Data analysis was performed using GraphPad Prism 9.3.1 (471; 2365 Northside Dr. Suite 560, San Diego, CA 92108, USA).

## Results

### T lymphocyte subsets in response to the immune-stimulatory molecules

In the first step, we wanted to investigate the capacity of the platform factors to induce CD4^+^ and CD8^+^ subpopulations. Human PBMCs, isolated and placed at a concentration of 10^6^/ml, were first exposed to the platform factors for 24 h at 37°C and 5% CO_2_. After the 24 h postinfection (hpi), cells were harvested, washed, and the amount of PBMC subpopulations were identified through staining for surface markers (CD197, CD95, CD8, CD4, CD45RA, and CD3) in an incubation buffer, held for 30 min at room temperature. Post two buffer washes, cell assessment was executed utilizing a FACS Lyric flow cytometer (BD Bioscience, NJ, USA) followed by analysis. Preliminary gating (supplementary Fig. S7) was informed by forward and side scatter within the lymphocyte population, and analysis was performed on 100 000 acquired events for each sample.

**Fig. 1:**
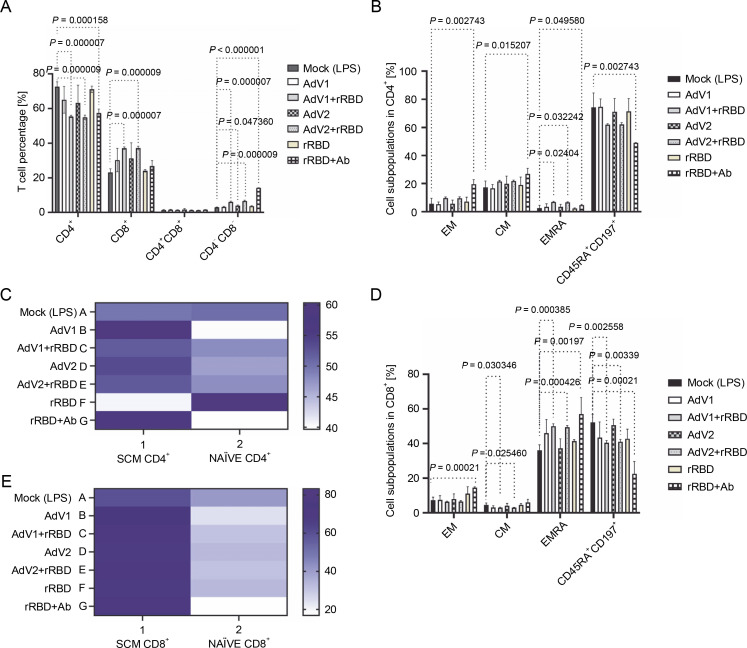
The percentage of the T lymphocyte subpopulations among PBMCs stimulated with the vaccine factors (*n* = 6) compared with PBMC mock (PBMCs stimulated with LPS at 1.25 μg/ml) after the 24-h treatment at 37°C in 5% CO_2_; (A) the percentage of CD4^+^ and CD8^+^ T among CD3^+^; (B) the percentage of central memory (CM), effector memory (EM), effector memory terminally differentiated (EMRA), and CD197^+^CD45RA^+^ among CD4^+^; (C) the heat map analysis of the markers expressed by the subpopulations of CD4^+^CD45RA^+^CD197^+^; the expression of CD95 (T_SCM_) after the treatment with AdV1 (left top row) or rRBD+Ab (left bottom row); the expression of NAÏVE (CD95^-^) after the treatment with rRBD (right bottom row); (D) CM, EM, EMRA, and CD45RA^+^CD197^+^ among CD8^+^; (E) The heat map analysis of markers expressed by the subpopulations of CD4^+^CD45RA^+^CD197^+^; the expression of CD95 (T_SCM_) after the treatment with AdV1 (left top row) or rRBD+Ab (left bottom row); the data are representative of 2–5 different experiments; multiple unpaired *t*-tests: only comparison with a *P*-value less than or equal to 0.05 was presented (significant diff. between the means of mock vs. the marked treatments, *P* ≤ 0.05)

We analyzed the CD4^+^T and CD8^+^T cell subset changes in pooled lymphocytes (Figs. 1A–E and supplementary Fig. S6). A notable alteration in the percentage of CD4^+^ cells in response to AdV1+rRBD was recorded at 55.4 ± 0.7% vs. mock 72.6 ± 2.8% (*P* = 0.000007) illustrated in Figure 1A. Conversely, the induction of CD8^+^T cells was roughly 37.2 ± 0.7% vs. mock at 23.1 ± 2.1% (*P* = 0.000007) in Figure 1A. Discernible differences (*P* < 0.015207) were identified between mock (2.6 ± 2.0%–74.2 ± 10.3%) and rRBD+Ab (4.6 ± 0.6%–49.0 ± 0.1%) across all CD4^+^T subpopulations (Fig. 1B). In the EMRA subpopulation, AdV1+rRBD elicited a more pronounced response than mock (6.9 ± 0.6% vs. 2.6 ± 2.0%, *P* = 0.024041). Moreover, AdV2+rRBD increased the EMRA subpopulations in PBMCs, markedly surpassing the levels in mock (6.6 ± 0.5% vs. 2.6 ± 2.0%, *P* = 0.032242). Furthermore, we observed amplified expression of CD4^+^T_SCM_ cells (59.8 ± 6.0% and 60.3 ± 2.7%) relative to mock (49.7 ± 5.0%, *P* = 0.3497) when exposed to AdV1 or rRBD+Ab (Fig. 1C). A surge in the percentage of TNaVve cells amongst CD4^+^ was also noted at about 59.4 ± 5.8% vs. mock (50.3 ± 6.3%, *P* = 0.3497) following rRBD stimulation.

Variations in the amount of CM, EM, EMRA, and CD45RA^+^CD197^+^ subpopulations among CD8^+^ (%) are depicted in Figure 1D. A significant increase (*P* = 0.00021) of CD8^+^T_EM_ for rRBD+Ab (14.5 ± 0.5%) vs. mock (7.2 ± 1.9%) was observed. The number of CD8^+^T_CM_ cells decreased after stimulation with AdV1+rRBD (3.0 ± 0.3%, *P* = 0.030346) or AdV2+rRBD (2.9 ± 0.2%, *P* = 0.025460) vs. mock (4.5 ± 1.2%). The increase in the number of CD8^+^T_EMRA_ cells was found after stimulation with AdV1+rRBD (50.1 ± 1.3%) , AdV2+rRBD (49.4 ± 1.0%), or rRBD+Ab (41.4 ± 0.8%) was recorded, compared to mock (36.0 ± 3.2%) (*P* < 0.00197). The CD45RA^+^CD197^+^ subpopulation of CD8^+^ decreased after stimulation with AdV1+rRBD (40.4 ± 1.3%, *P* = 0.0002558), AdV2+rRBD (41.0 ± 1.4%, *P* = 0.00339), or rRBD+Ab (22.4 ± 7.1%, *P* = 0.00021) vs. mock (52.2 ± 4.5%). An increased (*P* > 0.9999) number of CD8^+^T_SCM_ vs. mock was noted following all treatments (Fig. 1E).

A critical difference between CD4^+^ and CD8^+^ was the distinct expression of CD8^+^ T_EMRA_ (Fig. 1B vs. Fig. 1D) after the induction with AdV1+rRBD or AdV2+rRBD. Conversely, a decline was recorded in the number of CD8^+^CD45RA^+^CD197^+^ subpopulations after the stimulation with either AdV1+rRBD or AdV2+rRBD.

We discerned that rRBD+Ab (Fig. 1B, Fig. 1D) spearheaded the differentiation of T cells into various subpopulations: CD4^+^T_CM_ (26.7 ± 4.1%), CD4^+^/CD8^+^T_EM_ (19.5 ± 3.3%/14.5 ± 0.5%), CD4^+^/CD8^+^T_EMRA_ (4.6 ± 0.6%/57.0 ± 9.5%) vs. mock (17.4 ± 4.5%, 5.7 ± 3.9 %/7.2 ± 1.9%, 2.6 ± 1.9%/36.0 ± 3.2, respectively). Interestingly, no modifications in the CD8^+^ T_CM_ percentages were observed using rRBD+Ab, maintaining at 4.5 ± 1.2% vs. 6.0 ± 1.9% when compared to mock). Contrariwise, the number of CD4^+^/CD8^+^CD45RA^+^CD197 ^+^ subpopulations (49.0 ± 0.1%/22.4 ± 7.1%) were reduced vs. mock (74.2 ± 10.3%/52.2 ± 4.5%) after stimulation with rRBD+Ab.

### The B-cell subsets and IgD, IgM, and IgG index after the 24-h stimulation with immunogenic factors

We used flow cytometry to analyze the B cell subsets within pooled lymphocytes. A manual gating strategy and visualization of cellular data clusters for B-cell subpopulations demonstrated through a representative sample (FSC vs. SSC), are presented in supplementary Figure S7. The strategy included a selection of singlets, gating on CD19^+^, CD24^+^CD38^+^ transitional B cells, CD19^+^ CD24^-^CD38^+^ plasmablasts, CD38^-^CD24^+^CD27^+^CS, CD27^+^IgD^+^NCS, and IgD^+^ NAÏVE within memory B cells.

**Fig. 2:**
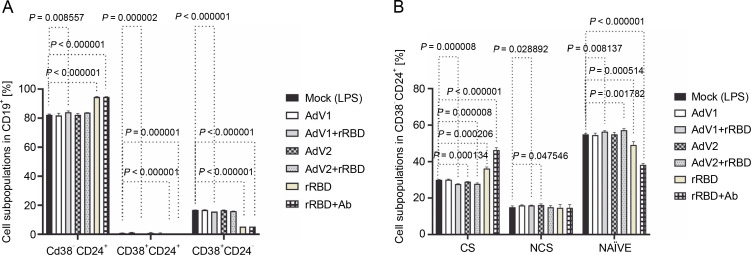
(A) the CD19^+^ lymphocyte subpopulations induced by the immunogenic factors after the 24-h treatment: CD38^-^CD24^+^ (memory cells), CD38^+^CD24^+^ (transitional cells), CD38^+^CD24^-^(plasmablasts); (B) the subsets of the memory B cells (CD19^+^ CD24^+^CD38^-^): CS, NCS, and NAÏVE; multiple unpaired t-tests: significant differences between the means of mock vs. the marked treatments (*P* ≤ 0.05). Data are shown as means ± SD of four independent experiments

Post a 24-h treatment with immunogenic factors, B cell phenotypes were extracted from PBMCs, followed by a harvesting, washing, and staining process with antibodies (CD24, CD38, CD19, IgD, CD27, and CD20) in an incubation buffer for 30 min at room temperature. The consequent changes within B cell subsets in pooled lymphocytes are illustrated in Figures 2A and 2B.

In detail, after coculturing PBMCs with the CD40 ligand-expressed AdV1+rRBD, we detected the CD19^+^ CD38^-^CD24^+^ memory B cells (84.1 ± 0.8% vs. 82.3 ±0.4%, *P* = 0.008557), the CD19^+^CD24^+^CD38^+^ transitional B cells (0.17 ± 0.05% vs. 0.9 ± 0.1%, *P* = 0.000002), and the CD19^+^CD38^+^CD24^-^ plasmablast B cells (15.7 ± 0.1% vs. 16.7 ± 0.1%, *P* < 0.000001) vs. mock in Figure 2A. The CD19^+^CD38^-^CD24^+^ memory B cells were the main expansion lymphocyte subset toward the immunogenic factors (Fig. 2A). The percentage of the memory B cells was about 94.6 ± 0.3% (*P* < 0.000001) or 94.6 ± 0.1% (*P* < 0.000001) vs. mock (82.3 ± 0.4%) after the treatment with rRBD or rRBD+Ab.

In subsets distinguished by their CD27 and IgD status (Fig. S8), CD27^+^ B cells—either class-switched (IgD^-^) or nonswitched (IgD^+^) memory B cells – alongside CD27^-^IgD^+^ NAÏVE B cells and a double-negative population (CD27^-^IgD^-^) with undefined properties similar to memory B cells were assessed. Figure 2B illustrates a significant decrease in CS cells within the CD19^+^ CD24^+^CD38^-^ Subpopulation post-treatment with AdV1+ +rRBD (27.7 ± 0.2%) or AdV2+rRBD (27.8 ± 0.5%) vs. mock (30.0 ± 0.2%) (*P* = 0.000008). A significant increase (*P* < 0.000206) in CS was found after the stimulation with rRBD (36.2 ± 0.9%) or rRBD+Ab (46.3 ± 1.3%) vs. mock (30.0 ± 0.2%). In the NCS cells, AdV1+rRBD (15.9 ± 0.2%) or AdV2 (16.2 ± 0.6%) induced response as compared to mock (15.0 ± 0.7%).

Moreover, NAUVE B cells exhibited an increase posttreatment with AdV1+rRBD (56.3 ± 0.5%) or AdV2+rRBD (57.2 ± 0.9%) compared to mock (49.1 ± 1.9%) (*P* < 0.008137). However, a decrease was noted posttreatment with rRBD (49.1 ± 1.9%) or rRBD+Ab (38.2 ± 0.9%) vs. mock (49.1 ± 1.9%) (*P* < 0.000514).

**Fig. 3:**
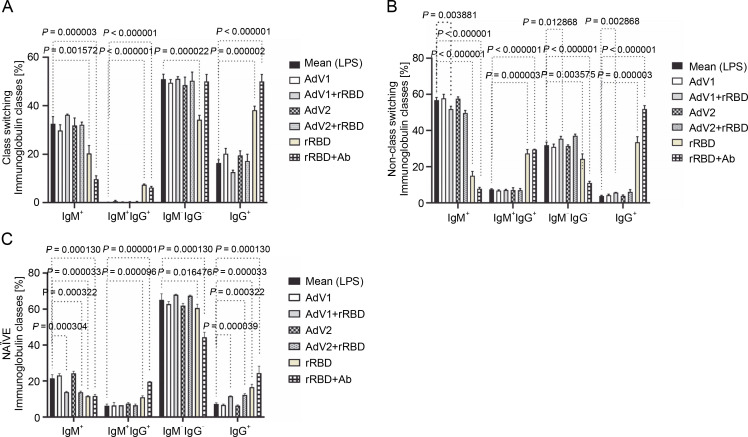
The immunoglobulin classes after the treatment with the immunogenic factors; (A) CD27^+^ and IgD^-^ allow the identification of class-switched B cells; (B) CD27^+^ and IgD^+^ allow the identification of nonclass-switched B cells; (C) CD27^-^ and IgD^+^ are representative of NAÏVE B cells; data are shown as means ± SD of four independent experiments; multiple unpaired *t*-tests: significant differences between the means of mock vs. the marked treatments (*P* ≤ 0.05)

In the case of class-switched B cells (Fig. 3A), for IgM^+^ (20.3 ± 3.3% or 9.7 ± 1.4% vs. mock 32.5 ± 3.0%, *P* < 0.001572), IgM^+^IgG^+^ (7.4 ± 0.3% or 6.2 ± 0.5% vs. 0.1 ± 0.1%, *P* < 0.000003), and IgG^+^ (38.1 ± 1.7% or 50.0 ± 2.9% vs. 16.4 ± 1.3%, *P* < 0.000002), the number of cells expressing these immunoglobulins on the surface were significantly changed after treatment with rRBD or rRBD+Ab. The following nonclass switching profiles were recorded (Fig. 3 B): 1) the percentage of IgM^+^ was elevated after priming with rRBD (33.5 ± 2.4%), rRBD+Ab (51.8 ± 1.6 %) vs. mock (56.8 ± 1.5, *P* < 0.000001); 2) the percentage of IgG^+^IgM^+^ was elevated after priming with rRBD (27.3 ± 2.2%), rRBD+Ab (29.5 ± 2.0%) vs. mock (7.5 ± 0.3%; *P* < 0.000003); 3) the percentage of IgG^+^ was elevated after priming with AdV1+rRBD (5.7 ± 0.3%, *P* = 0.002868), rRBD (35.5 ± 3.1%, *P* < 0.000001), rRBD+Ab (51.8 ± 2.0%, *P* < 0.000001) vs. mock (3.8 ± 0.4%). The distribution of IgG^+^ in NAÏVE B cells was as follows: 16.6 ± 1.6 % (*P* < 0.000033) or 24.4 ± 3.9% (*P* = 0.000130) after treatment with rRBD or rRBD+Ab vs. mock (7.2 ± 0.7) in Figure 3C. The gating strategy has been shown in supplementary Figure S8.

### Detection of soluble cytokines using flow cytometry

**Fig. 4:**
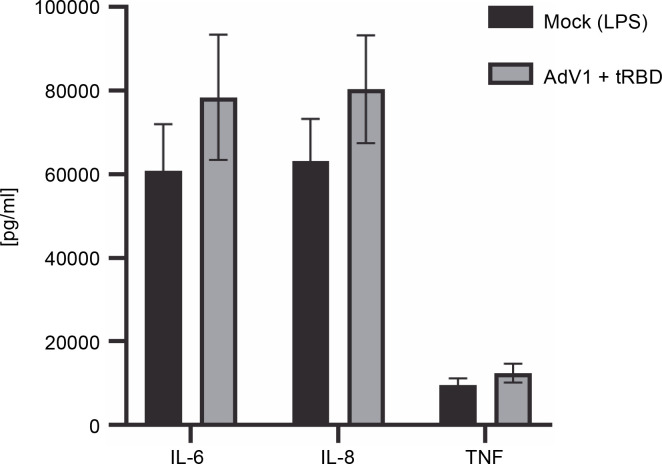
Effects of the AdV1+rRBD challenge on pro-inflammatory cytokines IL-6, IL-8, and TNF-α production in PBMC; PBMCs were treated with AdV1+rRBD for 24 h, and the supernatants were harvested for flow cytometry analysis; data are presented as means ± CV% (*n* = 6); TNF – tumor necrosis factor; IL-6 – interleukin 6; IL-8 – interleukin 8; PBMCs’ supernatant treated with LPS (Mock); PBMCs’ supernatant treated with AdV1: mixture of adenovirus 1 (AdV1, Ad5/3-D24 ICOS CD40L) at 100 VP/ml and rRBD at 2.62 μg/ml; Wilcoxon matched-pairs signed rank test: insignificant differences between the means of mock (LPS) vs. AdV1+rRBD (*P* = 0.25)

The cytometric bead array was performed to measure the secretion of 12 cytokines (IL-1 alpha, IL-1 beta, IL-2, IL-4, IL-6, IL-8, IL-10, IL-12p70 , IL-17A, IL-17F, TNF alpha, INF gamma) 24 h after immunization with AdV1+ + rRBD (Fig. 4). We detected IL-6 (78,470.43 pg/ml ± CV% 19.15 vs. 60849.14 pg/ml ± CV% 18.42), IL-8 (80388.44 pg/ml ± CV% 16.10 vs. 63182.36 pg/ml ± ± CV% 16.02), and TNF alpha (12445.88 pg/ml ± CV% 18.29 vs. 9504.45 pg/ml ± CV% 17.94). Contrariwise, the levels of the remaining cytokines were as follows: INF-γ (744.7 pg/ml ± CV% 20.95 vs. 1083.66 pg/ml ± CV% 20.95), IL-1α (109.27 pg/ml ± CV% 16.20 vs. 139.15% ± ± CV% 15.24) and IL-1 beta (343.35% ± CV% 18.14 vs. 336.8% ± CV% 17.73), IL-2 (64.44% ± CV% 20.27 vs. 66.69% ± CV% 17.14), IL-4 (no level detected), IL-10 (835.30% ± CV% 18.74 vs. 964.21% ± CV% 19.22), IL-12p70 (676.87% ± CV% 18.97 vs. 697.79% ± CV% 17.41), IL-17A (1.45% ± CV% 34.76 vs. 1.30% ± CV% 25.15), and IL-17F (18.51% ± CV% 29.14 vs. 33.99% ± ± CV% 24.64).

### Effects of the immunogenic factors on the CD40 expression in vitro

**Fig. 5:**
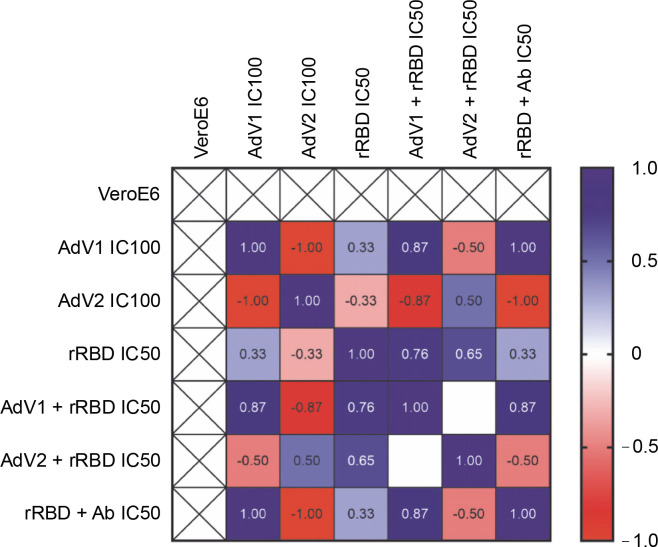
Relative quantification of the CD40 gene of VERO E6; values are given as the cycle threshold (Ct, mean of triplicate samples); normalization factors were calculated as the geometric mean of the expression levels of the most stable reference gene, GAPDH; a control VERO E6 sample was used as the calibrator (= 1); fold gene expression 2^-ΔΔCt^ was calculated according to the formula: ΔΔCt = ΔCt (sample) ˗ ΔCt (control average) and ΔCt = Ct (gene of interest) – Ct (housekeeping gene); gene expression values from all experiments are displayed in a heatmap indicating the up-regulation (blue) or downregulation (red) of given markers; interpretation of relationship strength between variables: *r*>1 (strongly positive), 0.5 < *r* < 1 (moderately positive), 0 < *r* < 0.5 (weakly positive), *r* = 0 (none), and negative correlation for the opposite direction; blank results mean the same value of the variable in the rows. the cell expression

To examine the effects of VERO E6 function activation under immunogenic factor conditions, real-time RT-qPCR was used to assess the expression of CD40 following 24 h with or without stimulation. CD40, a cell surface receptor protein, plays a pivotal role in the immune system, notably in the adaptive immune response. The stimulation of CD40 expression on the platform resulted in consistent effects on corresponding mRNA levels (Fig. 5). As shown in Figure 5 by the navy blue color, the CD40 gene was significantly upregulated when cells were stimulated with the following factors, compared to the untreated control: 1) AdV1 IC100 (100 VP/ml), 2) rRBD IC50 (2.62 μg/ml), 3) AdV1 + rRBD IC50 (50 VP/ml + 2.62 μg/ml), and 4) AdV2 + rRBD IC50 (50 VP/ml + 2.62 μg/ml). A strong positive correlation between AdV1 and rRBD was observed in CD40 upregulation (*r* > 1). The upregulation of the CD40 receptor allows immune cells to become more responsive to signals from T cells expressing CD40L, thus suggesting its involvement in the immune response to vaccination.

**Fig. 6:**
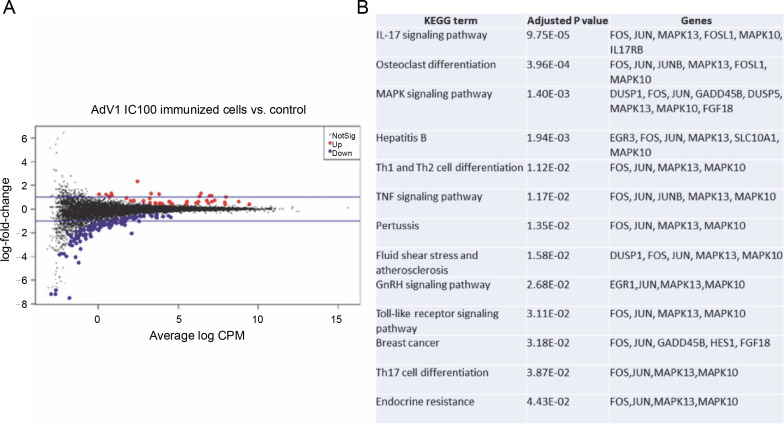
Gene expression changes – log-intensity ratios (differences) versus log-intensity averages (means) (A) and enrichment of up and down regulated genes in KEGG term gene groups (B) in AdV1 IC100 immunized cells; in A NotSig means not significantly expressed vs. mock, Up means expressed at a higher level compared with mock, and Down means expressed at a lower level compared to mock

The effects of VERO E6 function activation on wholecell expression under immunogenic factor conditions were determined via RNA-seq. The sequencing reads were demultiplexed using Illumina bcl2fastq (version 2.20). Skewer performed. For samples prepared with the Takara kit, the initial three nucleotides of the second sequencing read, originating from the Pico v2 SMART adapter, were trimmed using Skewer (version 0.2.2). In paired-end sequencing, read 2 corresponded to the sense strand. FastQC (version 0.11.5-cegat) by Andrews was used to assess the quality of FASTQ files, and data visualization was conducted in R (version 4.0.4) using ggplot2 (Wickham, 2009). Sequencing libraries prepared following rRNA depletion, were sequenced on an Illumina NovaSeq 6000 platform, generating 2 × 100 bp reads. The sequencing company executed adapter trimming and eliminated nucleotides with Phred scores below a specified threshold, retaining only reads exceeding 35 nucleotides in length. Alignment to the human GRCH.38 genome, with GENCODE version 43 primary annotation, was executed using the RSEM-1.3.3 coupled with the STAR-2.7.10b pipeline. Differential expression analysis utilized estimated read counts per gene with the DESeq2-1.34.0 R package. Notably, sample nr 8 under the PBMC AdV1+S+N condition exhibited outlier characteristics, significantly deviating from all PBMC cell samples and displaying nearly double the read count compared to other samples. Hierarchical clustering, employing the complete linkage method, was applied to these samples. Transformed read counts were calculated using the R dist() function with default parameters, based on the Euclidean distance between samples. The DESeq2 model encompassed all conditions, resulting in three separate comparisons. Differentially expressed genes were subsequently subjected to gene ontology enrichment analysis using GPrifiler or the DAVID tool, with the threshold for Benjamini–Hochberg adjusted FDR applied to identify significant gene expression differences in each comparison. Both upregulation and downregulation were noted in each condition with the highest number of differentially expressed genes relative to mock (adjusted *P* < 0.1) observed following AdV1 IC100 immunization (158 genes, Fig. 6A; supplementary File Excel with lists of genes). These genes are enriched in several KEGG terms such as IL-17, MAPK, TNF, GnRH, Toll-like receptor signaling pathways, and Th1, Th2, and Th17 cell differentiation (Fig. 6B). Raw RNA-seq fastq files and count files are available from the GEO repository under accession number GSE245876.

## Discussion

To our knowledge, we presented the first *in vitro* study utilizing an adenoviral platform to deliver T cell immunogens ICOS and CD40 ligands in formulation with rRBD. A PubMed search conducted on 15 January 2023, employing the terms: (SARS-CoV-2) and (vaccine) and (clinical trials) showed that vaccines administered intramuscularly have induced systemic antibody and T-cell responses (Hayward et al., 2015; Davis, 2020; Madhavan et al., 2022). It is well known that the innate immune system plays a fundamental role in determining the direction of the adaptive immune response and in sensing vaccines and adjuvants (Pulendran and Ahmed, 2011; Di Pasquale et al., 2015; Afkhami et al., 2016; Černý and Stříž, 2019; Meraviglia et al., 2019). Although *in vitro* neutralization assays are widely utilized and offer convenience, they cannot replace *in vivo* studies (preand clinical) in evaluating new viral vectors (Kiener et al., 2018). Our findings suggest that, despite the lack of cytotoxicity (supplementary Figs. S11–S14), the immunogenicity of the AdV platform in the *in vitro* study was not sufficient, prompting the need for further testing of the described combination as vaccine candidates in relevant *in vivo* models.

Initially, we carried out a phenotypic and functional analysis of CD4^+^T and CD8^+^T cells (Fig. 1) that stimulated a response in the context of vaccines against infection. These subpopulations not only control initial infections but also promote and maintain adaptive T-cell responses. CD4^+^T cells can minimize the severity of pulmonary lesions induced by viruses (Rouse and Sehrawat 2010). In our study, the responding CD8^+^T cells gained the ability to differentiate into TEMRA (50.0 ± 1.3% vs. 36.0 ± 3.2%), when activated with AdV1+rRBD early in the immune response (day 2). The TEMRA cells modulate cellular homeostasis in reaction to stress generated by immunogenic factors (Callender et al. 2018; Sharma and Rudra 2018). The proliferation of CD8^+^T cells, while pivotal in controlling viral spread postinfluenza infection, does not come without a cost (Shane and Klonowski, 2014). In our study, cell proliferation was associated with slightly increased apoptosis within the highly dividing population (supplementary Figs. S9–S13 and supplementary Table S3). We showed here that PBMCs displayed the apoptotic profile (supplementary Fig. S10) typical of the senescent-associated secretory phenotype (SAPS) (Lopes-Paciencia et al., 2019). Aligning with Mullen et al. (2012), our studies suggest a functional role for the TEMRA cells, both contributing to disease pathogenesis and providing immune surveillance.

Previous studies (Davis, 2020; Moss, 2022) emphasized that the subpopulation of T cells plays a synergistic role in orchestrating the immune response. Interest is growing in the role of CD4^+^T cells in the context of antiviral therapy. It is known that CD4^+^T_NAUVE_ cells differentiate into any of several subsets of helper T cells with effector function that mediates protection against different pathogens (Pulendran and Ahmed, 2011). Our *in vitro* studies have described several futures, including CD4^+^T_CM_ (21.5 ± 0.8% vs. 17.6 ± 1.5%, *P* > 0.05), CD4^+^T_EM_ (9.7 ± 0.8% vs. 5.7 ± 3.9%, *P* > 0.05), CD4^+^T_SCM_ (52.1 ± 6.1% vs. mock 49.7 ± 5.0%, *P* > 0.9999) in response to the AdV1+rRBD platform. There was weak evidence of the platform-specific memory CD4^+^T cell generation. According to Moss (2022), T_SCM_ showed longterm maintenance, which attracted interest in these cells to prevent viral infection and proliferation. In line with the findings presented by Cencioni et al. (2021), the antiviral potential of T_SCM_ downregulates the expression of the programmed cell death protein 1 (PD1). We postulated that the T_SCM_ downregulation mechanism is typical of the platform, signaling T cell costimulator (ICOS) and hence prone to T cell activation and differentiation. The ligation of T cells with the ICOS ligand on AdV1 led to the release of proinflammatory signals, favored tissue damage, and infection control. The molecules, e.g., CD40, ICOS, and PD-1, regulate germinal center differentiation, affinity maturation, and longevity of the immune response (Pulendran and Ahmed, 2011). An early study (Meraviglia et al., 2019) pointed to the virus-specific CD4^+^T_SCM_ and CD8^+^ T_SCM_ cell response during primary *ex vivo* infection. We identified a distinct immune pattern showing a high percentage of the T_EMRA_ cell response within the CD8^+^T cells.

Ever since the role of regulatory B cells in modulating inflammatory T cell function through CD40/CD40L was evidenced (Mohib et al. 2020; Cencioni et al. 2021) highlighted, we sought to identify antigen-specific B cells after immunization. The *in vitro* distribution of memory (CD19^+^CD38^-^CD24^+^), NAÏVE, and transitional B cells in PBMCs stimulated with AdV1+rRBD were analyzed and found to contain a higher proportion of the memory B cell subset (Fig. 2). Following the previous results (Cencioni et al., 2021), we suggested that CD40 engagement typifies a model of T cell-dependent B cell activation. Evidence suggests that AdV1+rRBD may influence regulatory B cells. Consistent with Michel et al. (2017), we demonstrated that CD40 ligation activated mitogenassociated protein kinase (MAPK)-, phosphoinositide 3-kinase (PI3K)-, and nuclear factor-6B (NF-κB)-signaling events triggering subsequent proinflammatory gene expression (Fig. 6).

We showed (Fig. 5) that the CD40 gene, regulated by inflammatory stimuli, including TNF-α and CD40L (Hassan et al., 2009), was significantly expressed in VERO E6 in a manner inducible following stimulation with platform factors. According to several studies (Danese et al., 2004; Néron et al., 2006; Elgueta et al., 2009), CD40 ligation resulted in cell survival, proliferation (supplementary Figs. S11–S14), and the expression of proinflammatory cytokines (Fig. 5 and supplementary Fig. S5A–M). Upon CD28 signaling, cytokine expression levels, such as IL-6 and IL-8 (Fig. 5), were found to increase in T cells. CD28 engagement has been shown to enhance the expression of costimulatory molecules, including CD40 (Michel et al., 2017).

In our study, B cells transitioned from producing IgM and IgD to isotype IgG upon stimulation with platform factors (Fig. 3A). As the class-switching process is modulated by cytokines (Bonilla and Oettgen 2010), we observed that IL-6 and IL-8 (Fig. 4) promoted switching to IgG. In line with the results obtained by other authors, Bonilla and Oettgen (2010), the primary response of B lymphocytes was found to trigger the production of lower-affinity IgM antibodies (Fig. 3A).

Through RNA-seq, several KEGG pathways were identified as enriched within genes differentially expressed by VERO E6 cells after stimulation with the AdV1+ +rRBD platform. Notably, these pathways encompass Th1, Th2, and Th17 cell differentiation gene expression patterns, each playing a vital role in providing protection against different pathogens (Pulendran and Ahmed, 2011) . The RNA-seq helped to determine the signaling pathways that control the immunological mechanisms by which the AdV1+rRBD platform evokes protective immunity. These include the MAPK cascade, adipocytokine, cAMP, TNF, and Toll-like receptor (TLR) pathways.

TLR activation catalyzes a cascade via at least two distinct pathways, culminating in the production of both proinflammatory cytokines, such as TNF, and anti-inflammatory cytokines such as IL-6 (Grassin-Delyle et al., 2020). Furthermore, the activation of the TNF signaling pathway can elicit a wide range of effects, extending from cell proliferation to apoptosis (Grassin-Delyle et al., 2020). The combination of AdV1+rRBD induced IL-6, IL-8, and TNF alpha, compared with the mock (*P* = 0.25), also stimulating IgM, IgG, and altering the CD4^+^T/CD8^+^T ratio. *In vitro*, neither mouse nor human IgM exhibited direct neutralizing activity against AdV (Allen and Byrnes, 2019). In contrast, IgG disrupted steps within the cell entry process (Allen and Byrnes, 2019). Moreover, both IL-6 and TNF have showcased vaccine adjuvant activity (Talaat et al., 2018). The preclinical and clinical development of AdV-based vaccines against tuberculosis underscored the significance of Th1 and CD8^+^T-cell responses, mediated through TNF alpha (Afkhami et al., 2016).

Whole transcriptome sequencing of VERO E6 cells revealed differential expression of several apoptosis genes upon stimulation with the AdV1+rRBD platform (Fig. 6A and Fig. 6B). Moreover, flow cytometry not only identified but also quantified apoptotic hallmarks (supplementary Fig. S11 and supplementary Table S3). Given that apoptosis can be triggered by external environmental changes, such as activation by small TNF (Talaat et al., 2018), we observed higher levels of TNF upon platform stimulation. Immunogenic factors compromising plasma membrane integrity were also demonstrated (supplementary Fig. S11 and supplementary Table S3).

The VERO E6 cells’ relative expression profile suggested an interaction between epithelial cells and cytotoxic T lymphocytes, notably through the MAPK axis and Jun N-terminal kinase (Fig. 6B). Memory CD4^+^T and CD8^+^T cells, detected in recovered patients, affirmed that protective memory T cells can form following *in vitro* immunization. In our experiment, rRBD and neutralizing rRBD Ab induced memory CD4^+^/CD8^+^T cells and memory B cells with an IgG class-switching subset (Figs. 1–3). Developing a vaccine that stimulates B cells to produce SARS-CoV-2 specific Abs is paramount to providing viral prevention and protection (Chen et al., 2022). Our *in vitro* data support the results of clinical studies showing a protective role for the cell responses primed by various vaccines like hAd-S-Fusion+ETSD (CD4^+^Tcells), VXA-CoV2-1 (CD8^+^T cells), and ChAd-SARS-CoV-2/BBV154 (CD8^+^T cells) (Mendonça et al., 2021) . Moreover, clinical data for other viral vectored (e.g., Convidecia, AdVOVID, Ad26.COV2-S, Sputnik V, GRAd-COV2, ChAdOX1-nCoV) and mRNA vaccines (Pfizer-BioNTech and Moderna) have shown capabilities in inducing both humoral and cellular immunity (Mendonça et al., 2021). Our analysis provided insight into the generation of protective immune memory cells in an *in vitro* model utilizing human PBMCs to assess the adenoviral platform vaccine. The role of these platformspecific T cells’ capacity to protect from a future infection remains to be further determined, particularly considering prolonged stimulation periods within the 3D culture model.

## Conclusion and limitation of the study

We have reported the memory T-cell responses subsequent to immunization with the AdV1+rRBD platform, noting a composition of resultant CD8^+^T cells that included CD8^+^T_EMRA_ and CD8^+^T_SCM_ cells, which demonstrated notable cytotoxicity. Therefore, we expected that the approach would be effective for obtaining T-cell-specific prevention against infectious diseases. The proposed adenoviral platform generated the ability of the system to record its experience with SARS-CoV-2 and rapid responses to subsequent challenges with the same infection.

However, whether these results will translate to an *in vivo* vaccination context remains speculative. Additionally, our *in vitro* 2D model features a relatively short course of immunization (24 h). Thus, the effects of the immunogenic factors on SARS-CoV-2 prevention in both 3D and *in vivo* models. Despite AdV being the focal point of these *in vitro* studies, further investigations are crucial to comprehend whether NAb-mediated inflammation persists *in vitro/in vivo* scenarios. Furthermore, the low immunogenicity observed with the AdV platform indicates avenues for further studies, potentially exploring the administration of vectors at higher concentrations.

## Competing interests

The authors declare that they have no competing interests.

## Authors’ contributions

J.B., Ł.K., M.G., and M.S. carried out immunogenicity studies. M.S. and L.K. were responsible for the conceptualization of the studies. M.T. was responsible for SEM analyses. A.M.P., D.M., and A.M. conducted molecular genetics studies related to rRBD and editing the manuscript. T.S., M.Ł., and D.P. were responsible for bioinformatic analyses and RNAseq data presentation. The manuscript was reviewed by L.K., M.W., K.P., E.H., L.B., T.C., J.M., and M.S. M.S. was responsible for financing the studies. All authors read and approved the final manuscript.

## Supplementary Material

*In vitro* immune evaluation of adenoviral vector-based platform for infectious diseases
