# Supplementary material for: In vitro immune evaluation of adenoviral vector-based platform for infectious diseases
Source: BioTechnologia (Pozn). 2023 Dec 21;104(4):403–19. doi: 10.5114/bta.2023.132775 (PMC10777723; doi:10.5114/bta.2023.132775)
Supplement: In vitro immune evaluation of adenoviral vector-based platform for infectious diseases [file BTA-104-4-51760-s001.pdf]

BioTechnologia vol. 104 (4) · pp. 403–419 · 2023
Journal of Biotechnology, Computational Biology and Bionanotechnology SUPPLEMENTARY MATERIALS
https://doi.org/10.5114/bta.2023.132775

CC BY 4.0

In vitro immune evaluation of adenoviral vector-based platform for infectious diseases

JOANNA BARAN[1], ŁUKASZ KURYK[2], TERESA SZCZEPIŃSKA[1], MICHAŁ ŁAŹNIEWSKI[1], MARIANGELA GAROFALO[3], ANNA MAZURKIEWICZ-PISAREK[1], DIANA MIKIEWICZ[4], ALINA MAZURKIEWICZ[1], MACIEJ TRZASKOWSKI[1], MAGDALENA WIECZOREK[2], KATARZYNA PANCER[2], EWELINA HALLMANN[2], LIDIA BRYDAK[2], DARIUSZ PLEWCZYNSKI[5,6], TOMASZ CIACH[4], JOLANTA MIERZEJEWSKA[7], MONIKA STANISZEWSKA[1]*

[1] Centre for Advanced Materials and Technologies, Warsaw University of Technology, Warsaw, Poland

[2] National Institute of Public Health, Warsaw, Poland

[3] University of Padova, Padova, Italy

[4] Faculty of Chemical and Process Engineering, Warsaw University of Technology, Warsaw, Poland

[5] Faculty of Mathematics and Information Science, Warsaw University of Technology, Warsaw, Poland

[6] Centre of New Technologies, University of Warsaw, Warsaw, Poland

[7] Faculty of Chemistry, Warsaw University of Technology, Warsaw, Poland

Received: 3 April 2023; revised: 12 September 2023; accepted: 29 September 2023

Abstract

New prophylactic vaccine platforms are imperative to combat respiratory infections. The efficacy of T and B memory cell-mediated protection, generated through the adenoviral vector, was tested to assess the effectiveness of the new adenoviral-based platforms for infectious diseases. A combination of adenovirus AdV1 (adjuvant), armed with costimulatory ligands (ICOSL and CD40L), and rRBD (antigen: recombinant nonglycosylated spike protein rRBD) was used to promote the differentiation of T and B lymphocytes. Adenovirus AdV2 (adjuvant), without ligands, in combination with rRBD, served as a control. *In vitro* T-cell responses to the AdV1+rRBD combination revealed that CD8+ platform-specific T-cells increased (37.2 ± 0.7% vs. 23.1 ± 2.1%), and T-cells acted against SARS-CoV-2 via CD8+TEMRA (50.0 ± 1.3% vs. 36.0 ± 3.2%). Memory B cells were induced after treatment with either AdV1+rRBD (84.1 ± 0.8% vs. 82.3 ± 0.4%) or rRBD (94.6 ± 0.3% vs. 82.3 ± 0.4%). Class-switching from IgM and IgD to isotype IgG following induction with rRBD+Ab was observed. RNA-seq profiling identified gene expression patterns related to T helper cell differentiation that protect against pathogens. The analysis determined signaling pathways controlling the induction of protective immunity, including the MAPK cascade, adipocytokine, cAMP, TNF, and Toll-like receptor signaling pathway. The AdV1+rRBD formulation induced IL-6, IL-8, and TNF. RNA-seq of the VERO E6 cell line showed differences in the apoptosis gene expression stimulated with the platforms vs. mock. In conclusion, AdV1+rRBD effectively generates T and B memory cell-mediated protection, presenting promising results in producing CD8+ platform-specific T cells and isotype-switched IgG memory B cells. The platform induces protective immunity by controlling the Th1, Th2, and Th17 cell differentiation gene expression patterns. Further studies are required to confirm its effectiveness.

Key words: adenoviral vectors; vaccine platform; innate and adaptive immunity

* Corresponding author: Centre for Advanced Materials and Technologies, Warsaw University of Technology, 02-822 Warsaw, Poland; e-mail: Monika.Staniszewska@pw.edu.pl

***Gold Coated Sample Preparation for SEM Imaging* (Hitachi SU8230, Japan)**

The monolayers of NCI-H226 cells grown on glass slides (ř 9 mm) in 24-well plates were separately infected with the adenoviruses. Then, after 24-h incubation samples were primarily fixed using glutaraldehyde (5% v/v in phosphate buffer 0.1M, pH 7.2; Sigma-Aldrich), and secondary fixed with 1% OsO_4 (Sigma-Aldrich). The fixed specimens were dehydrated by incubation in a series of ethanol increased gradually without causing specimen shrinkage (35%, 50%, 75%, 95% v/v in water). The specimens were mounted on metal stubs using carbon adhesive discs. The specimens were coated with gold using Quorum 150TS sputter coater and analyzed using the Hitachi SU 8230 Field Emission Scanning Electron Microscope (FE-SEM) at 8 mm working distance and 10.0 kV landing voltage.

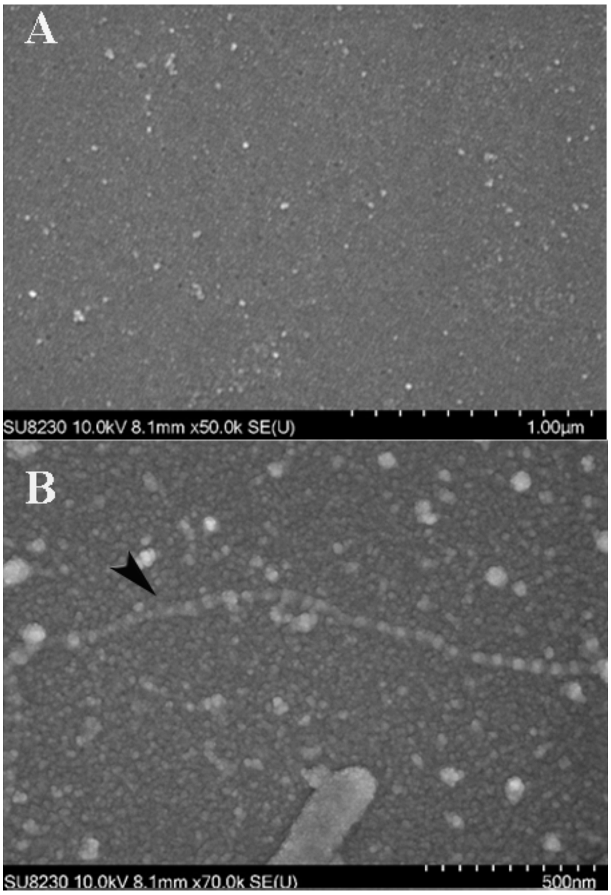

Fig. S1. Scanning electron micrograph SEM. (A) Morphology of glass slides. (B) Adenovirus-infected cells presented a discreet increment in the number of microvilli with the MOIs of 100 VP/ml of medium. Adenovirus marked the arrowhead.

Table S1. The titer of Adenovirus AdV5.3-d24-E3 in NCI-H226 growing in the ATCC-formulated RPMI-1640 Medium (ATCC USA) supplemented with fetal bovine serum (ATCC, USA) to a final concentration of 10%. ATCC (LGC Standard, Lomianki, Poland) supplemented with 1% of penicillin/streptomycin (Gibco Laboratories, USA) and 10% fetal bovine serum (FBS, Gibco Laboratories, USA). CPE was documented using an inverted microscope (×400 mag.)

Spearman-Kärber plate
Cell line: NCI-H226
Virus name: AdV5.3 -d24-E3

dilution		1	2	3	4	5	6	7	8	9	10	11	12
-1	A	+	+	+	+	+	+	+	+	X	-	-	-
-2	B	+	+	+	+	+	+	+	+	X	-	-	-
-3	C	+	+	+	+	+	+	+	+	X	-	-	-
-4	D	-	-	-	-	-	-	-	-	X	-	-	-
-5	E	-	-	-	-	-	-	-	-	X	-	-	-
-6	F	-	-	-	-	-	-	-	-	X	-	-	-
-7	G	-	-	-	-	-	-	-	-	X	-	-	-
-8	H	-	-	-	-	-	-	-	-	X	-	-	-
											Control		

Legend: log TCID50 = L-d(S-0.5), where L = log of lowest dilution used in the test; d = the difference between log dilution steps; and S = the sum of the proportion of "positive" tests (i.e., cultures showing CPE). Viral stocks and collected samples were titrated by tissue culture infectious dose 50% (TCID50 ml−1) in the NCI-H226 cells, using the Kärber formula. $logID_{50}$=log(dilution giving highest CPE) – log(dilution factor) × (Σ infected rate at each dilution – 0.5); $logID_{50}$=-3-1*(8/8+8/8+8/8-0.5)=-5.5; 1/10^-5.5/0.1=3162278; 3.2x10^6 $TCID_{50}$/ml

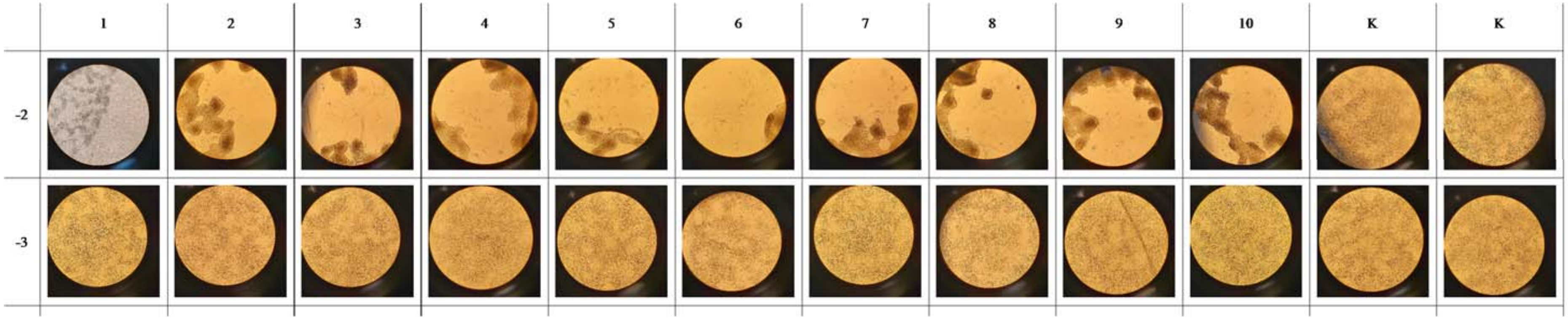

Fig. S2. Adenovirus 5/3 D24-ICOS-CD40L (extinction coefficient of 1.1 × 10^{12}/ Abs 260 unit) infected NCI-H226 cell cultures growing in ATCC-formulated RPMI-1640 Medium (ATCC USA) supplemented with fetal bovine serum (ATCC, USA) to a final concentration of 10%. (columns from 1 to 10 in rows marked -2 and -3). A lack of morphological changes was noted in the cell cultures in a row (-3) in columns (1-10) vs control (K). CPE was assessed through daily observation of infected cultures vs control (K). CPE: swelling and clumping of cells were observed. Infected cells grow and clump together in “grape-like” clusters. Detachment (death) of the cells in the monolayer was noted. Uninfected cultures distinguish normal cell changes that occur as cells age. Inverted light microscope (×100 mag.). $logID_{50}$=log(dilution giving highest CPE) – log(dilution factor)x(Σ infected rate at each dilution – 0.5); $logID_{50}$=-2-1*(10/10-0.5)=-2.5; 1/10^-2.5/0.1=3162.278; 3.2×10^3 $TCID_{50}$/ml

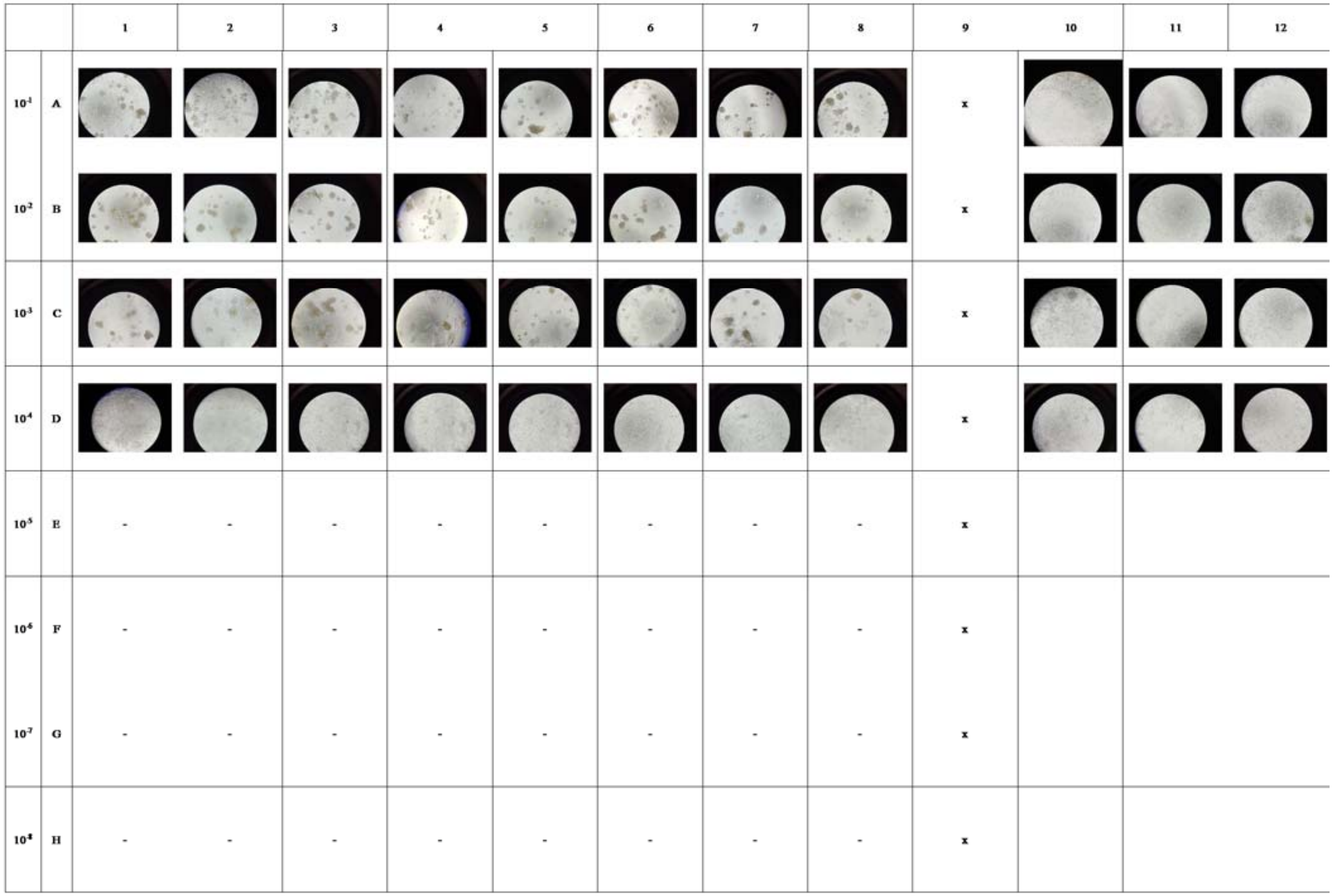

Fig. S3. AdV5.3-d24-E3 (extinction coefficient of 7.7×10[12]/ Abs 260 unit) infected NCI-H226 cell cultures growing in ATCC-formulated RPMI-1640 Medium (ATCC USA) supplemented with fetal bovine serum (ATCC, USA) to a final concentration of 10%. (columns from 1 to 10 in rows marked -2 and -3). A lack of morphological changes was noted in the cell cultures in a row (-4) in columns (1-8) vs control (K). CPE was assessed through daily observation of infected cultures vs control (K). CPE: swelling and clumping of cells were observed. Infected cells grow and clump together in “grape-like” clusters. Detachment (death) of the cells in the monolayer was noted. Uninfected cultures distinguish normal cell changes that occur as cells age. Inverted light microscope (×400 mag.)

Table S2. Sequence of rRBD based on:

Source: https://www.ncbi.nlm.nih.gov/nuccore/1798174254<br><br>/organism="Severe acute respiratory syndrome coronavirus 2"<br>/isolate="Wuhan-Hu-1"<br>/host="Homo sapiens"<br>/country="China"<br>/collection_date="Dec-2019"
a. Amino acid sequence GenBank: QHD43416.1<br><br>5' Met + RBD + FT4 + SerGlySer + 6HisTag + STOP 3' (232 aa = 696 bp)<br><br>MNITNLCPFGE VFNATRFASV YAWNRKRISN CVADYSVLYN SASFSTFKCY GVSPTKLNDL CFTNVYADSF VIRGDEVRQI APGQTGKIAD YNYKLPDDFT GCVIAWNSNN LDSKVGGNYN YLYRLFRKSN LKPFERDIST EIYQAGSTPC NGVEGFNCYF PLQSYGFQPT NGVGYQPYRV VVLSFELLHA PATV GYIPEAPRDG QAYVRKDGEW VLLSTFL SGSHHHHHH+ STOP
b. nucleotide sequence (codon usage *E.coli*)<br><br>5' ATG AAC ATT ACC AAC CTG TGC CCG TTC GGC GAA GTG TTC AAC GCG ACC AGA TTC GCG TCT GTG TAT GCG TGG AAC CGT AAA CGT ATT TCT AAC TGC GTG GCG GAT TAT TCT GTG CTG TAT AAC TCT GCG TCT TTT TCT ACC TTT AAA TGC TAT GGC GTG TCT CCG ACC AAA CTG AAC GAT CTG TGC TTT ACC AAC GTG TAT GCG GAT TCT TTT GTG ATT CGT GGC GAT GAA GTG CGT CAG ATT GCG CCG GGC CAG ACC GGC AAA ATT GCG GAT TAT AAC TAT AAA CTG CCG GAT GAT TTT ACC GGC TGC GTG ATT GCG TGG AAC TCT AAC AAC CTG GAT TCT AAA GTG GGC GGC AAC TAT AAC TAT CTG TAT CGT CTG TTT CGT AAA TCT AAC CTG AAA CCG TTT GAA CGT GAT ATT TCT ACC GAA ATT TAT CAG GCG GGC TCT ACC CCG TGC AAC GGC GTG GAA GGC TTT AAC TGC TAT TTT CCG CTG CAG TCT TAT GGC TTT CAG CCG ACC AAC GGC GTG GGC TAT CAG CCG TAT CGT GTG GTG GTG CTG TCT TTT GAA CTG CTG CAT GCG CCG GCG ACC GTG GGC TAT ATT CCG GAA GCG CCG CGT GAT GGC CAG GCG TAT GTG CGT AAA GAT GGC GAA TGG GTG CTG CTG TCT ACC TTT CTG TCT GGT TCT CAT CAC CAT CAT CAC CAT TAA 3'

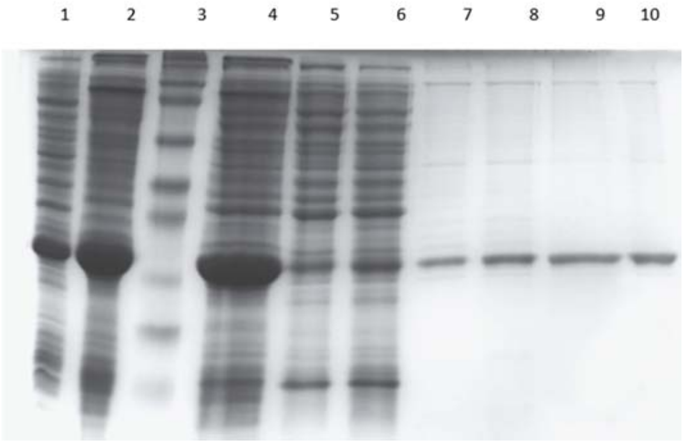

Fig. S4. SDS-PAGE analysis of rRBD expression in *E. coli* and purification on NiNTA Sepharose SuperFlow chromatography

Legend: Proteins were separated in 15% acrylamide gel and stained by Coomassie Brilliant Blue. Lanes: 1 - Lysate of *E. coli* transformed with pT7/RBD plasmid; 2 – Bacterial cell pellet after sonication; 3 - Protein molecular marker (12.0-225.0 kDa) (Full-Range Rainbow, Amersham, UK); 4 - Dissolved inclusion bodies (50 mM phosphate buffer, 5 mM β-mercaptoethanol, 7 M urea pH 12.0); 5 - Protein unbound to NiNTA Sepharose column; 6 - Fractions after washing (50mM phosphate buffer pH-7.0, 7 M urea, 300 mM NaCl, 25 mM imidazole); 7,8,9 - Fractions eluted from NiNTA Sepharose column (50 mM phosphate buffer pH 7.0, 7 M urea, 300 mM NaCl, 300 mM imidazole); 10 - rRBD protein after elution and 24h dialysis (50 mM phosphate buffer pH 8.0, 10% glycerol).

Flow cytometry FACS Lyric flow cytometer (BD Bioscience, NJ, USA)

Cell apoptosis was tested according to Annexin V Staining Protocol (BD) recommending 10× Binding Buffer (cat. no. 556454): 0.1 M HEPES, pH 7.4; 1.4 M NaCl; 25 mM CaCl2; Propidium Iodide (PI, cat. no. 556463) uses in parallel with Annexin V-FITC (cat. no. 556420). Cells were washed twice with cold 1× PBS Buffer (cat. no. 554781): 8 g NaCl, 0.2 g KCl, 1.44 g Na_2HPO4, $7H_20$, 0.24 g KH_2PO4, H_20 to 1 liter (adjusted pH to 7.2, autoclaved and store at room temp.) and then resuspend cells in 1× Binding Buffer at a concentration of ~1 × 10^6 cells/mL. Then 100 μl of the cell solution (~1 × 10^5 cells) was transferred to a 5 ml culture tube and Annexin V and Vital Dye. After gently mixing the cells and incubating for 15 min at room temp. in the dark, 1× Binding Buffer at 400 μl was added to each tube. Cells were analyzed by flow cytometry within 1 h.

Standard Samples of Quantitative Analysis of Cytokines

Plex Components

		Analyte		
Name	Lot Number	Name	Model	2nd Reporter
E7		Human IFN-γ	Quantitative	No
D6		Human IL-1α	Quantitative	No
B4		Human IL-1β	Quantitative	No
A4		Human IL-2	Quantitative	No
A5		Human IL-4	Quantitative	No
A7		Human IL-6	Quantitative	No
A9		Human IL-8	Quantitative	No
B7		Human IL-10	Quantitative	No
E5		Human IL-12p70	Quantitative	No
B5		Human IL-17A	Quantitative	No
C6		Human IL-17F	Quantitative	No
D9		Human TNF	Quantitative	No

Fig. S5A. Human IFN gamma standard curve

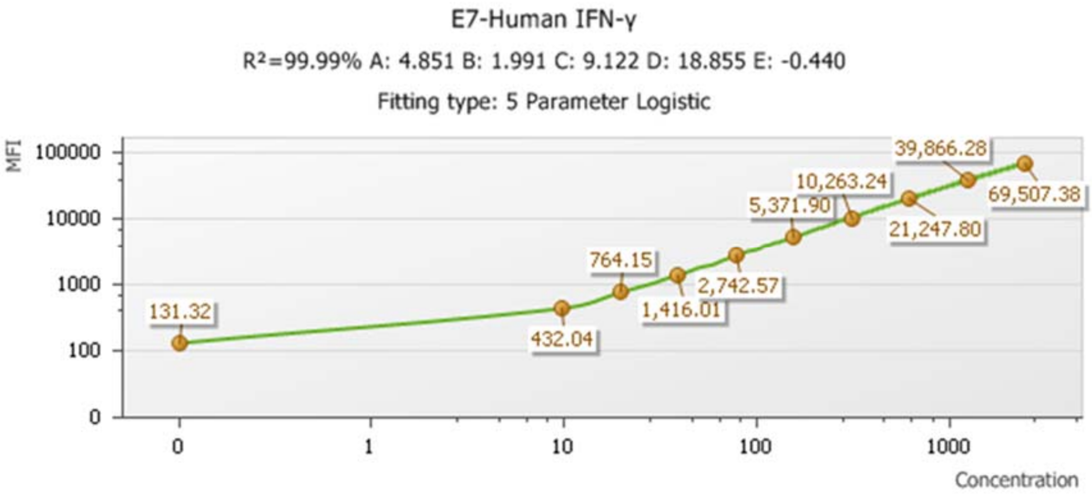

Fig. S5B. Human IL-1 alpha standard curve

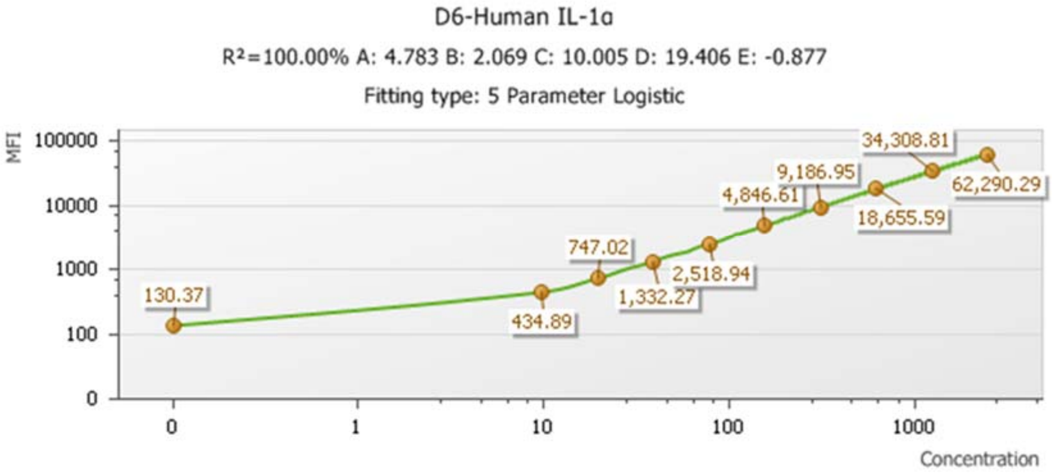

Fig. S5C. Human IL-4 standard curve

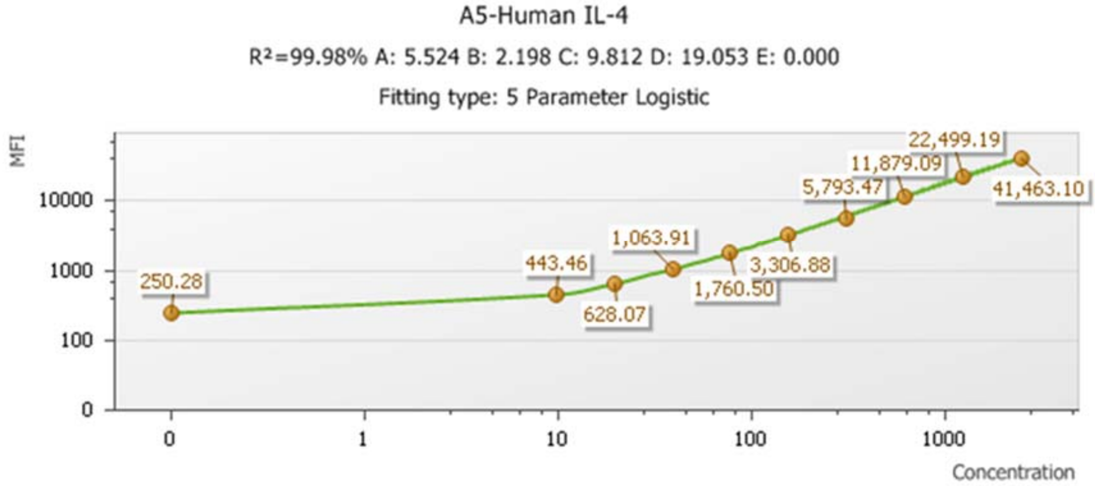

Fig. S5D. Human IL-6 standard curve

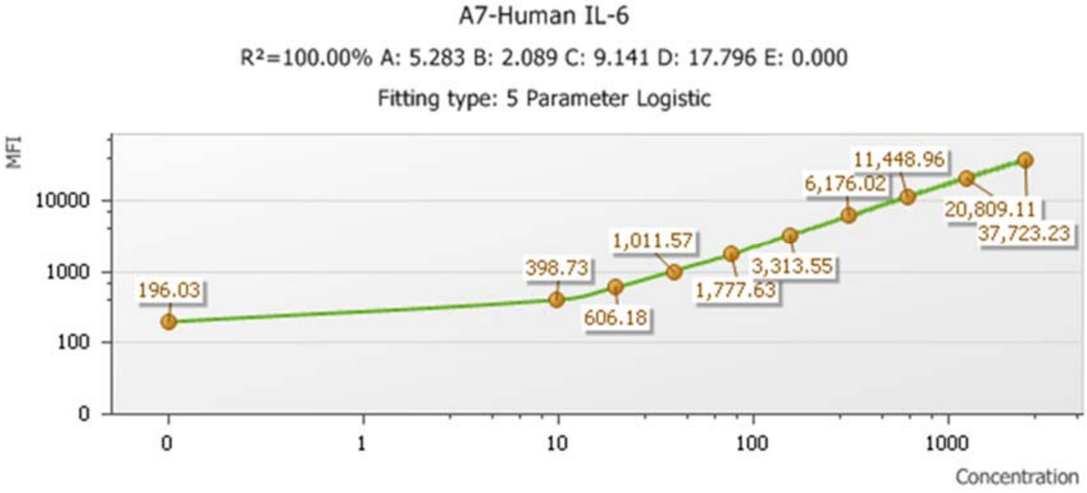

Fig. S5E. Human IL-2 standard curve

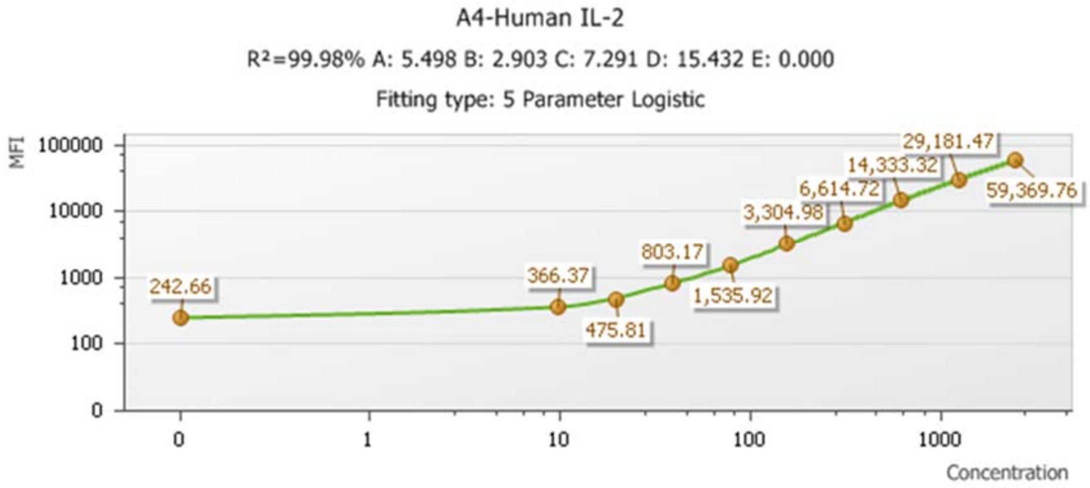

Fig. S5F. Human IL-1 beta standard curve

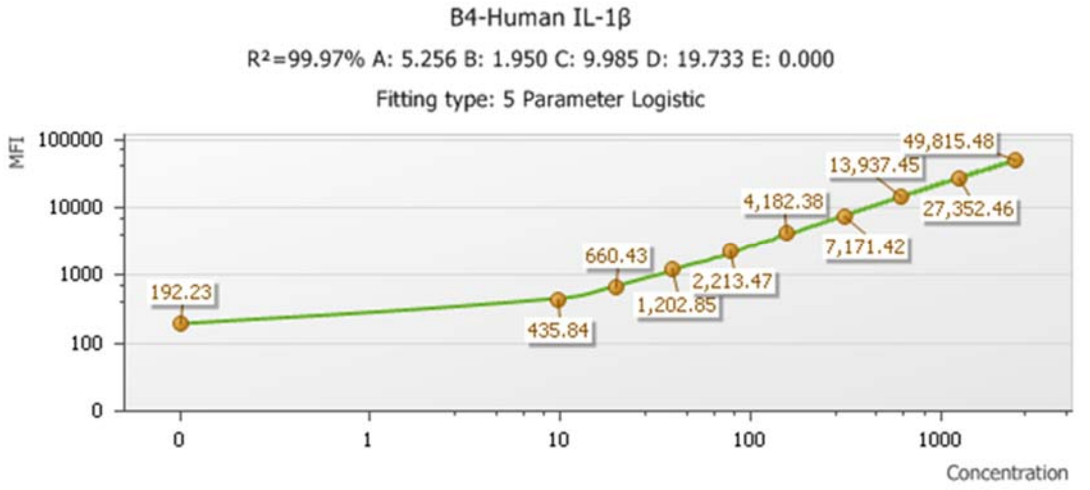

Fig. S5G. Human IL-8 standard curve

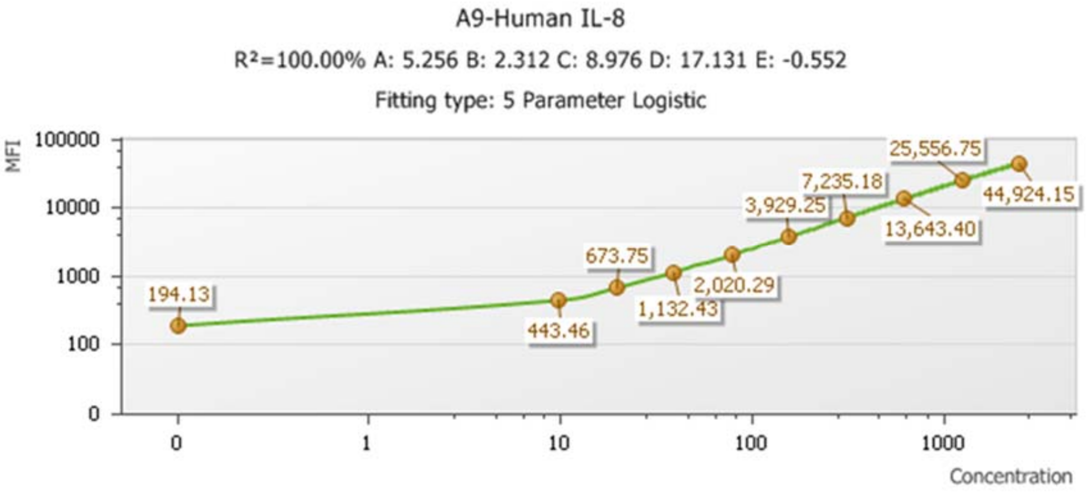

Fig. S5H. Human IL-12p70 standard curve

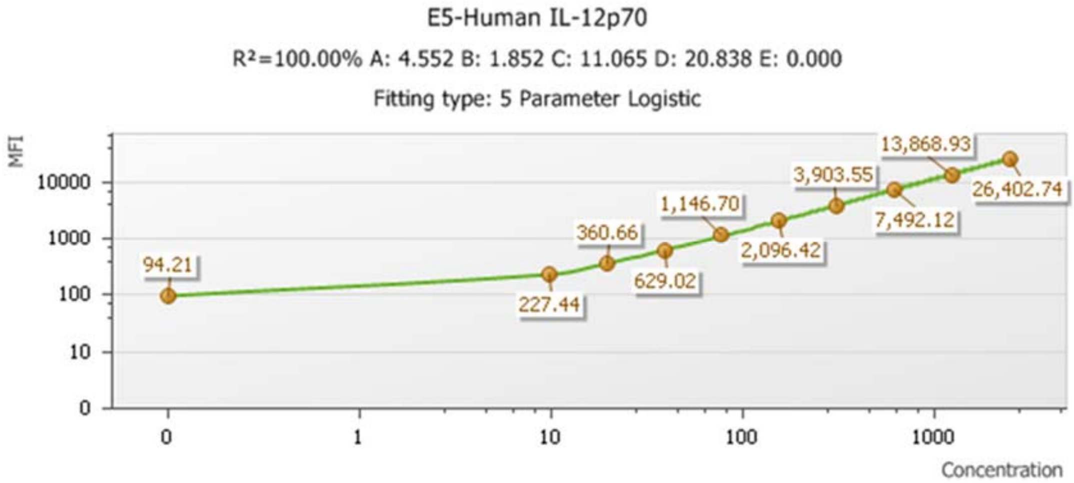

Fig. S5I. Human IL-10 standard curve

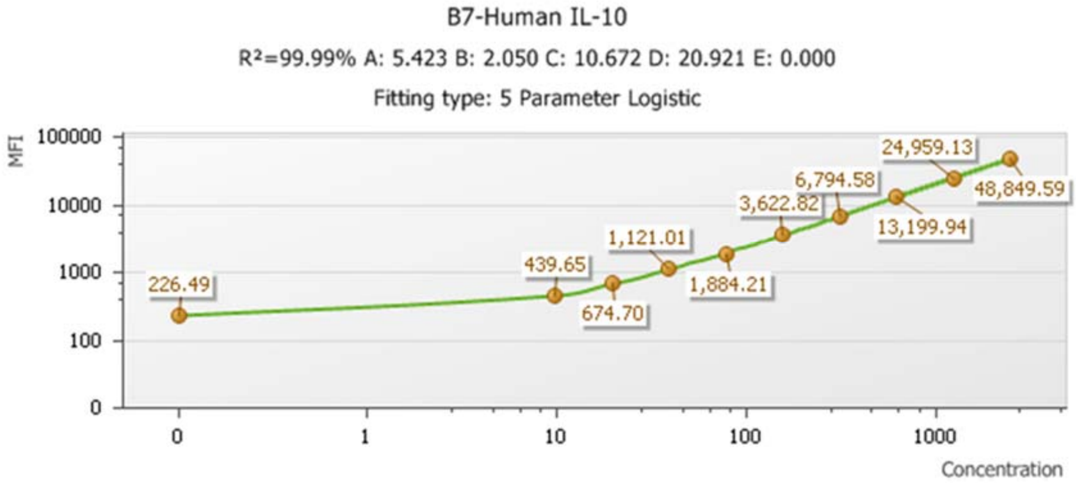

Fig. S5J. Human IL-17F standard curve

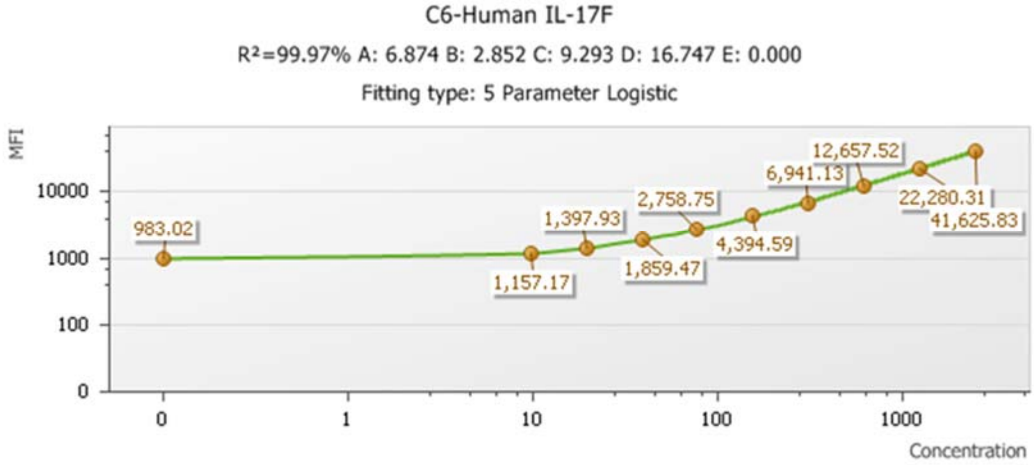

Fig. S5K. Human IL-17A standard curve

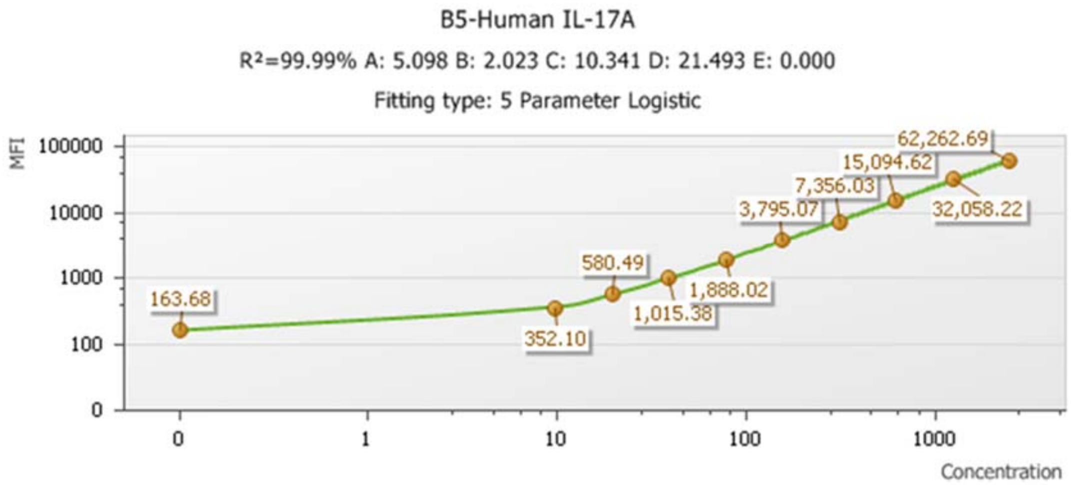

Fig. S5L. Human TNF standard curve

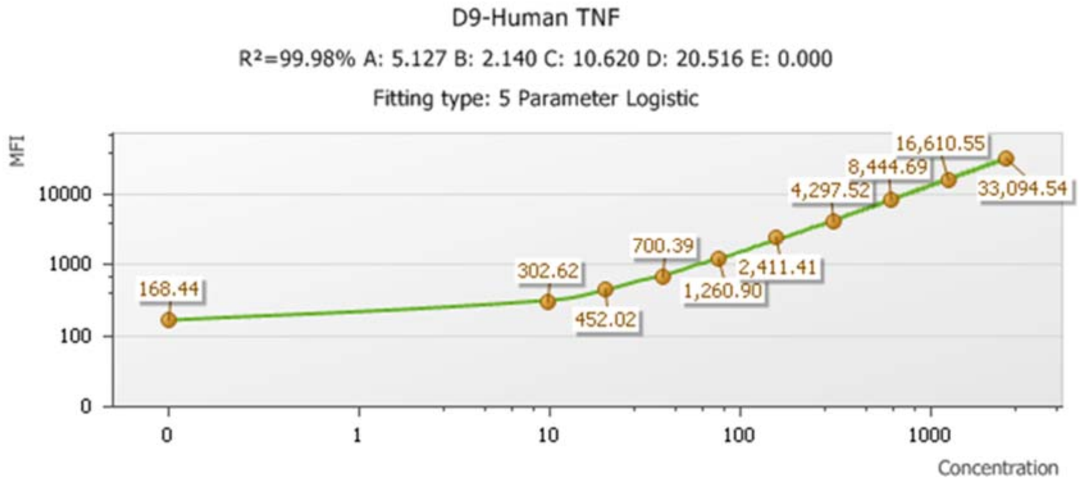

Fig. S5M. Gating strategy for CBA flex analysis

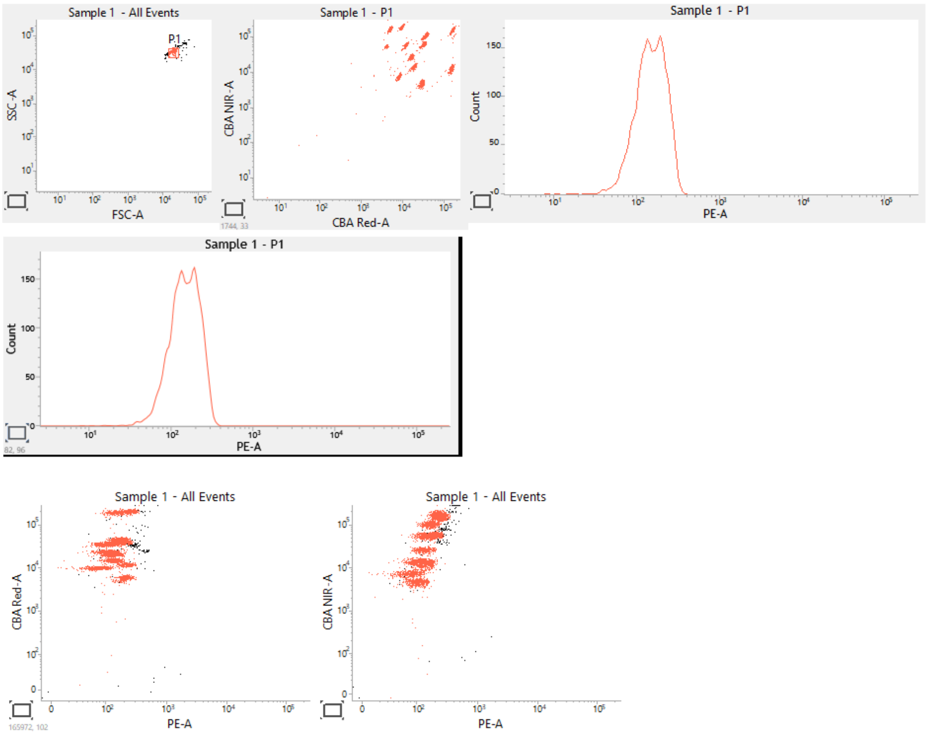

Determination of the HoV-OC43 and AdV Virus Particles Using UV Absorbance

Determination of the virus particle count was performed by UV spectrophotometry (Tecan, Männedorf, Switzerland) (Porterfield and Zlotnick 2010). The virus was suspended in the lysis buffer (1 M TRIS-EDTA, 10% SDS; pH = 7) at ratios of 1:3 (suspension of VP: lysis buffer), 1:5, 1:10, 1:50, and 1:100. The samples were incubated at 95 °C for 15 min, centrifuged briefly, and preserved on ice. We evaluated the VP in solution correlating to RNA content, quantified using a Spark microplate reader (Tecan, Männedorf, Switzerland). The UV absorbance was measured at 260 nm for viral RNA content and 280 nm for protein content. Furthermore, RNA purity was judged as 260 nm/280 nm = 2.0. The viral particle concentration was calculated using the method described by Maizel et al. (1968). The extinction coefficient was 1.1×10^{12} viral particles per OD 260 unit. We calculated VP using Equation (3): $VP = A260 \times \text{dilution factor} \times 1.1 \times 10^{12}/\text{ml}$, (3) where the 260 nm/280 nm ratio was 2.0 and the absorbance at 260 nm was 0.1–1.0 OD unit. 6.09×10^{11} VP/ml.

The gating strategy for the lymphocyte population by forward and side scatter.

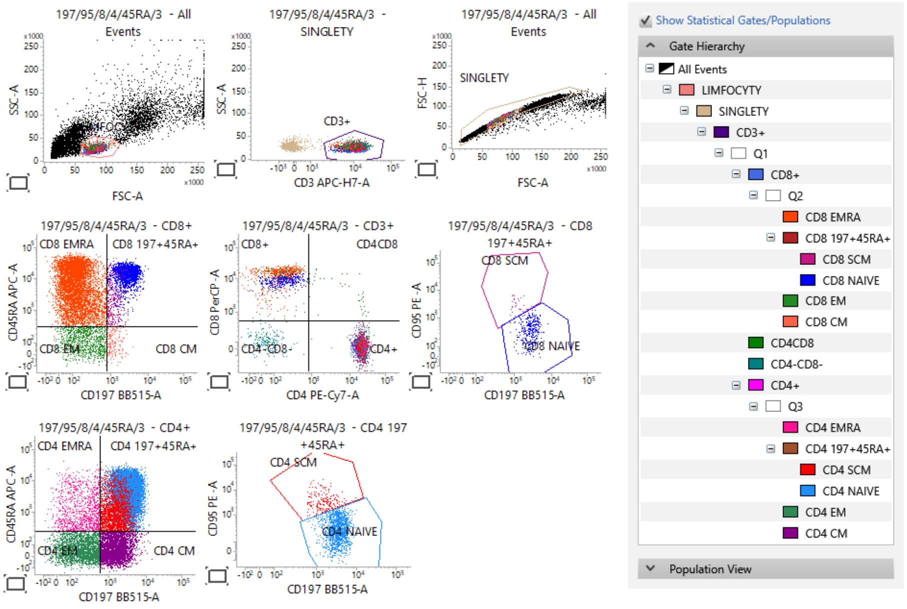

Fig. S6 Gating strategy for lymphocyte $CD4^{+}$ and $CD8^{+}$.

Legend: Manual lymphocyte gates were set in the light to scatter histograms (forward scatter versus side scatter, FSC vs. SSC) and T lymphocyte subpopulations were analyzed in the respective fluorescence histograms. Data were analyzed as follows: selection of singlets (named SINGLETY); gating on CD45+RA leukocytes; gating on CD3+ T-cells; gating on CD4+ and CD8+ strategy for analysis of lymphocyte subpopulations: EMRA, EM, CM, and gating on CD4+ CD197+CD45RA+ and CD8+ CD197+CD45RA+ for analysis of SCM, NAĎVE.

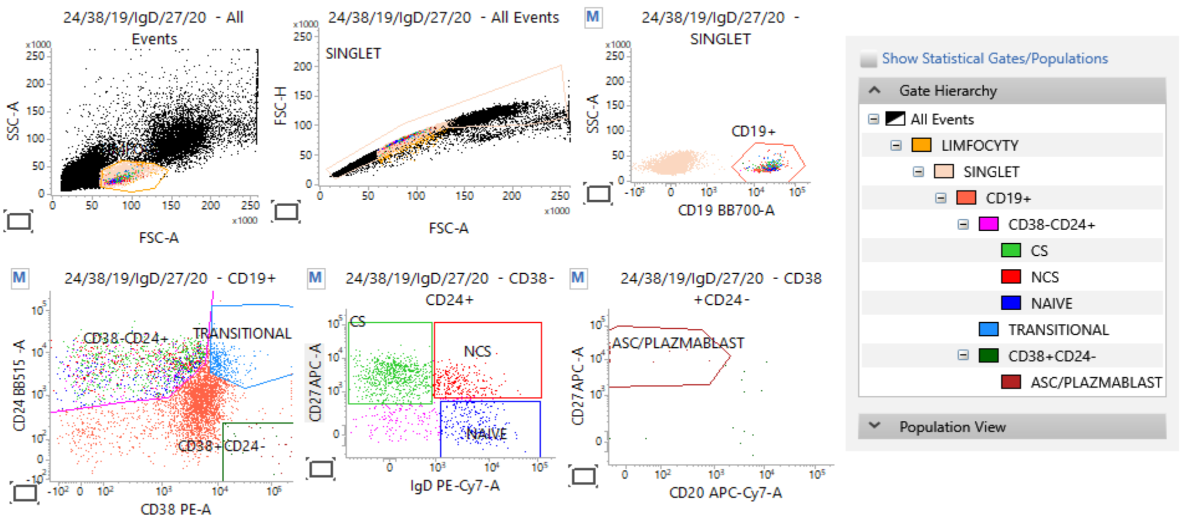

Fig. S7 Gating strategy for lymphocyte $CD19^{+}$.

Legend: Gating strategy of PBMC stained with CD19 BB700 (cat. no. 566396, BD USA), CD27 APC (cat. no. 561297, BD USA), CD38 PE (cat. no. 555460, BD USA), IgM BB515 (cat. no. 564622, BD USA), IgD (cat. no. PE-Cy7, BD USA), IgG APC-H7 (cat. no. 561297, BD USA). Flow cytometry analysis of the frequency of B cell subsets gated on CD19+B cells: CD19+CD38intCD24int naďve B cells (NAĎVE), CD19+CD38hiCD24hi transitional B cells (TRANSITIONAL), CD19+CD38-CD24hi memory B cells, and CD19+CD38hiCD24- plasma B cells

(ASC/PLAZMABLST). Flow cytometry plots show the CD27 (y-axis) and IgD status (x-axis) of PBMCs established with different factors. Manual gating strategy and visualization of the unbiased clustering of single-cell multidimensional data for the panel B-cell subpopulations on a representative sample (forward scatter versus side scatter, FSC vs. SSC): selection of singlets; gating on CD19+, gating on CD24+CD38+ transitional and CD19+CD24−CD38+ plasmablasts, gating on CD38-CD24+CD27+CS, gating on CD27+IgD+NCS, gating on IgD+ naďve in plasmablasts.

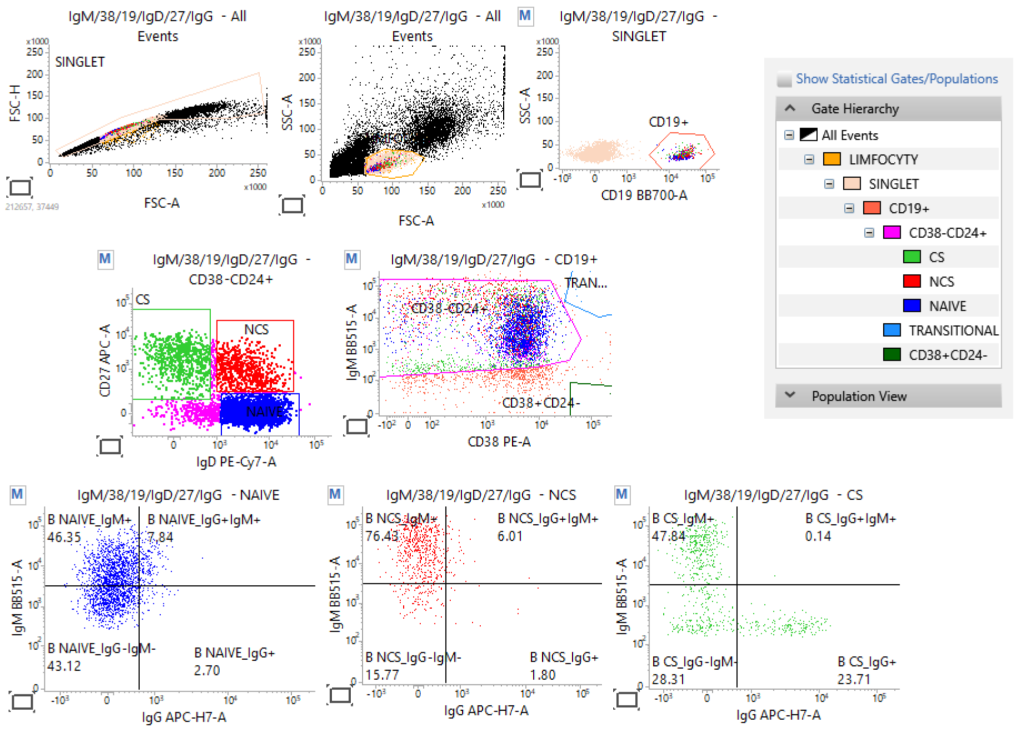

Fig. S8. Gating strategy – an example flow cytometry plot of B cells (CD19+) stained with CD27 and IgD

Legend: Characterization of B cells using the 6-color panel. PBMCs were stained with IgM (BB515, cat. no. 564622), CD38 (PE, cat. no. 555460), CD19 (BB700, cat. no. 566396), IgD (PE-Cy7, cat. no. 561314), CD27 (APC, cat. no. 558664), IgG (APC-H7, cat. no. 561297) to identify various B cell subsets. Colored boxes show the gates' position to separate the four subsets. IgD-/CD27- B cells (Magenta) are not a functionally defined subset (or could be non-B cells), so have not been named. The numbers in the plots show the percentage of cells in each quadrant.

Immunogenic factors' cytotoxicity toward PBMC and Vero E6

Cell metabolic activity assay

The PBMC and Vero E6 (ATCC USA) cells' metabolic activity was evaluated using a 3-(4,5-dimethyl-thiazol-2-yl)-2,5- diphenyltetrazolium bromide (MTT) assay kit (ab211091, Abcam Plc., Aibo Trading Co., Ltd., Shanghai, China) (Lu et al. 2021). The cells were seeded in triplicates at a concentration of 5×10^4 per well in Corning®Costar® 96-well plates (Sigma-Adrich Inc., St. Louis, MO, USA) containing 100 µl culture medium and allowed to adhere in a 5% CO_2 incubator at 37 °C overnight. Thereafter, the cells were treated with the immunogenic factors for 18 h. Then 20 µL of MTT solution was added to each well and incubated in a 5% CO2 incubator at 37 °C for 2 h. Next, optical densities (ODs) were measured at a wavelength of 490 nm using a Spark microplate reader (Tecan, Männedorf, Swiss).

Effects of Immunogenic factors on the PBMC and Vero E6 cytotoxicity and cell programmed cell death

The gating strategy for the programmed cell death using flow cytometry is presented in Fig. S10. We examined the effect of the immunogenic factors on the cytotoxicity to PBMC (1×10^4 cell/mL of Opti-MEM) (Fig. S11). In general, all tested factors were not toxic to PBMC after 24-h. Moreover, the Vero E6 cells were used due to the interactions of rRBD with the ACE2 receptor to gain entry into a cell to initiate infection (Madhavan et al. 2022). Only, rRBD at IC100=5.24 μg/mL displayed a cytotoxic effect against the VeroE6 cells (Fig. 10S-11S). AdV1 was not toxic to the VERO E6 cells at the tested concentration range (Fig. 12S). We next examined whether the immunogenic factors affect the programmed cell death of PBMC (Fig. 5B) and Vero E6 (Table S2) by assessing the cell membrane alternation using the flow-cytometric analysis of propidium iodide vs An-nexin V staining. The programmed cell death was assessed for PBMC cultured for 24 h with the immunogenic factors and then medium containing stimulators was removed and new restimulation till 7 days was performed. We did not observe any marked effects of single immunogenic factors on the PBMC viability, the treated cells were in the early apoptotic stage (Fig. 5 B). Contrariwise, the PBMCs stimulated with rRBD, AdV1+rRBD or AdV2+rRBD respectively showed apoptotic phenotypes (82.9-90.5±0.5% vs mock 47.22±0.6% ($p<0.0001$). The immunogenic factors induced apoptosis of the Vero E6 cells vs mock (Table S2, $p<0.0001$).

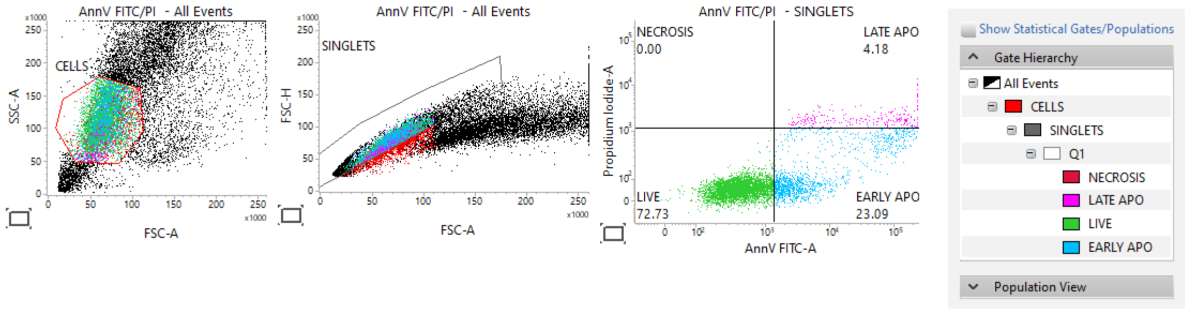

Fig. S9. Gating strategy for programmed cell death

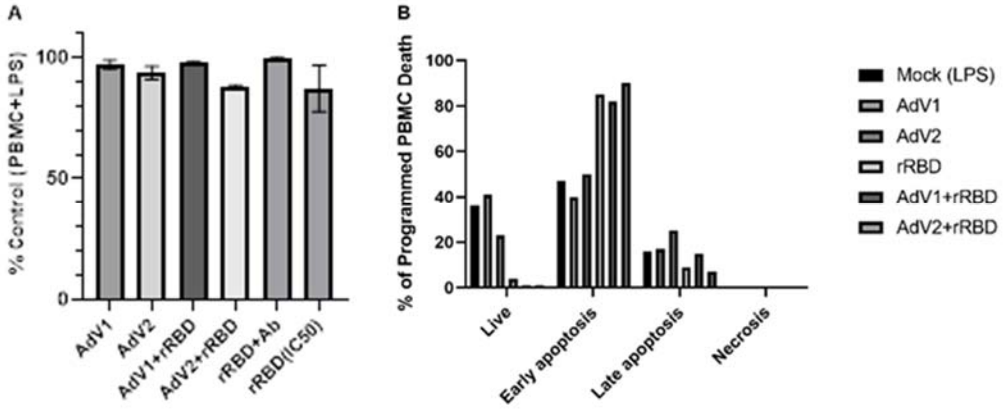

Fig. S10. PBMC proliferation and programmed cell death after the 24-h treatment with the immunogenic factors

Legend: (A) PBMC proliferation after the 24-h treatment with the immunogenic factors (no significant diff. among the means of treatments, $p>0.05$). (B) PBMC programmed cell death. Significant differences were noted between live cells and early apoptotic cells treated with rRBD, AdV1+rRBD or AdV2+rRBD ($p<0.00001$). Data are shown as means ± SD of three independent experiments.

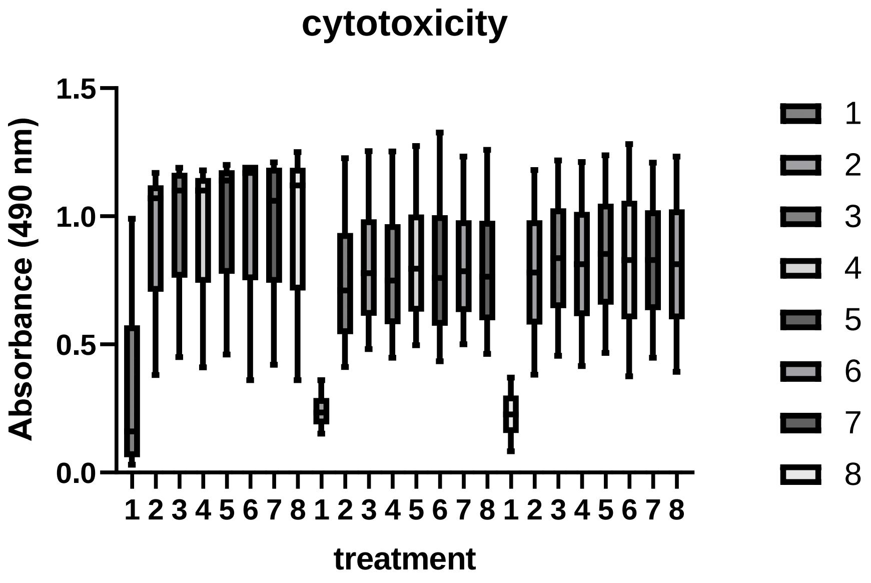

Fig. S11. The cytotoxicity of immunogenic factors to the VERO E6 cells (ATCC)

Legend: **1** means AdV1 at $5.2*10^{9}$ VP/ml+rRBD at 5.24 µg/mL; **2** means AdV1 at $5.2*10^{8}$ VP/ml+rRBD at 5.24*10 ^{1}µg/ml; **3** means AdV1 at $5.2*10^{7}$ VP/ml+rRBD at $5.24*10^{-2}$ µg/ml; **4** means AdV1 at $5.2*10^{6}$ VP/ml+rRBD at $5.24*10^{-3}$ µg/ml; **5** means AdV1 at $5.2*10^{5}$ VP/ml+rRBD at $5.24*10^{-4}$ µg/ml; **6** means AdV1 at $5.2*10^{4}$ VP/ml+rRBD at $5.24*10^{-5}$ µg/ml; **7** means AdV1 at $5.2*10^{3}$ VP/ml+rRBD at $5.24*10^{-6}$ µg/ml; **8** means control untreated.

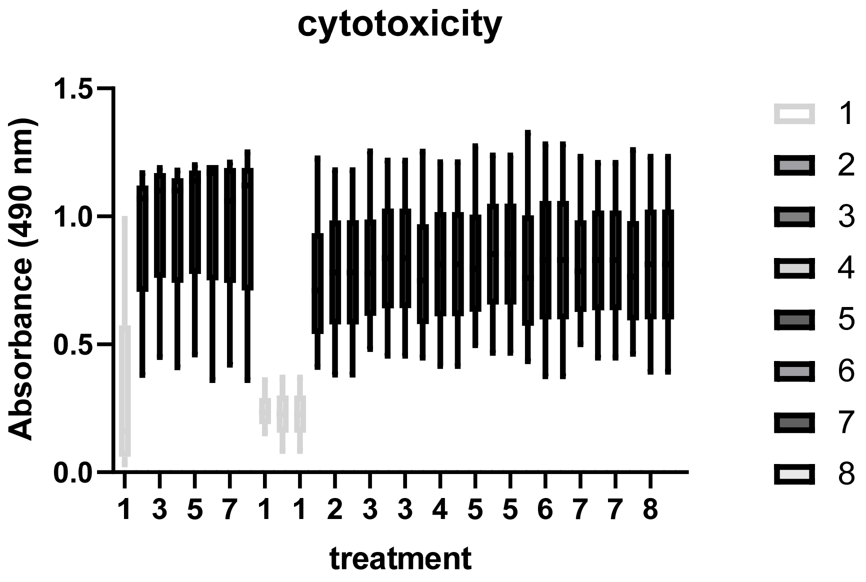

Fig. S12. The cytotoxicity of rRBD to the VERO E6 cells (ATCC)

Legend: **1** means rRBD at 5.24 µg/ml; **2** means rRBD at $5.24*10^{-1}$ µg/ml; **3** means rRBD at $5.24*10^{-2}$ µg/ml; **4** means rRBD at $5.24*10^{-3}$ µg/ml; **5** means rRBD at $5.24*10^{-4}$ µg/ml; **6** means rRBD at $5.24*10^{-5}$ µg/ml; **7** means rRBD at $5.24*10^{-6}$ µg/ml; **8** means untreated control of VERO E6.

Adenovirus 1 (AdV1) was not toxic to the VERO E6 at the tested concentration range (**Fig. S12**).

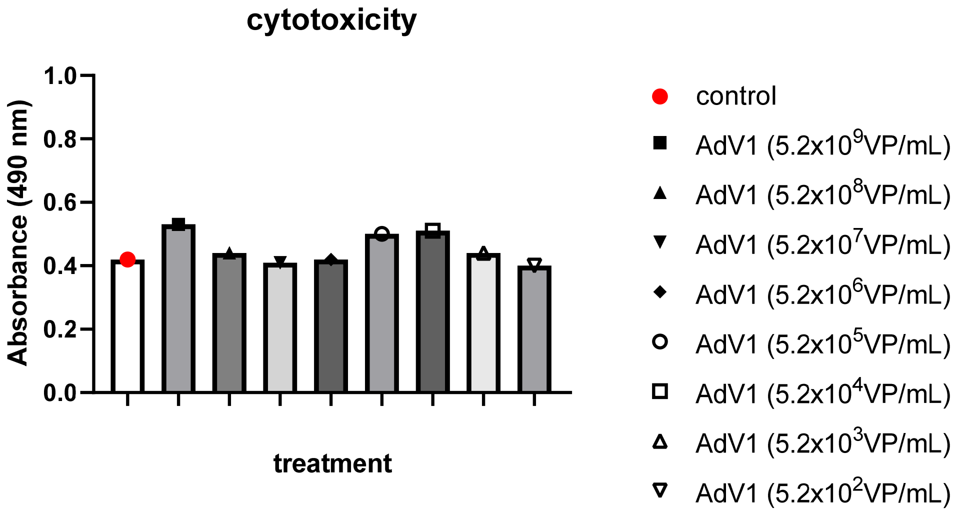

Fig. S13. The cytotoxicity of adenovirus (AdV1) to the VERO E6 cells (ATCC)

Based on the results (**Table S3**), a minimum two-fold reduction of the live cell counts vs the mock for each factor was observed. Apoptotic cells were generated under the influence of factors. Statistically significant differences between Vero E6 cells exposed to the vaccine platforms compared to mock were noted. For each sample, the number of cells in the necrosis phase was near zero.

Table S3. Mean results of the Vero E 6 programmed cell death (SD). Flow cytometry

Sample no.	Live cells		Early apoptotic cells		Late apoptotic cells		Necrotic cells	
	mean	SD	mean	SD	mean	SD	mean	SD
1	17.05	9.55	41.58	21.72	41.36	12.36	0.03	0.03
2	18.01	12.13	47.60	19.51	34.38	13.12	0.02	0.04
3	10.40	5.14	51.37	4.38	38.24	0.78	0.01	0.01
4	18.16	2.33	31.86	15.92	49.93	17.91	0.06	0.10
5	11.91	6.53	23.55	15.37	64.53	17.35	0.02	0.03
6	9.11	1.07	47.00	15.67	43.89	14.86	0.00	0.00
7	15.41	11.43	32.78	21.51	51.81	32.93	0.00	0.01
8	16.66	7.53	54.78	0.84	29.22	6.09	0.00	0.00
9	20.22	10.04	52.09	3.06	27.68	8.70	0.02	0.02
10	8.95	6.49	28.30	8.31	62.74	1.9	0.00	0.01
Mock	67.93	5.45	22.61	5.37	9.46	0.89	0.01	0.01

Legend: Sample 1 - Pseudovirus SARS-CoV-2 >100 RFU/ml; **Sample 2** – HCoV-OC43 200x diluted; **Sample 3** - Pseudovirus SARS-CoV-2 >100 RFU/ml, 'SARS-CoV-2 Spike Antibody' 5,24 µg/ml preincubated with SARS-CoV-2 for 24h; **Sample 4** - AdV I 100 VP/ml, rRBD 5,24 µg/ml; **Sample 5** - AdV I 100 VP/ml, rRBD 5,24 µg/ml, HoV-OC43 200x diluted; **Sample 6** - 100 VP/ml AdV I; **Sample 7** - 5,24 µg/ml rRBD; **Sample 8 -** 0,524 µg/ml rRBD. **Sample 9** - 100 VP/ml AdV I 0,524 µg/ml rRBD; **Sample 10** - 100 VP/ml AdV I, 0,524 µg/ml rRBD, 200x diluted OC43; Mock: (untreated Vero E6). **Abbreviations**: AdV I - Adenowirus Ad5/3-D24-ICOS-CD40L; VP – viral particles; rRBD – recombinant protein RBD; RFU – (Relative Fluorescence Unit).

References

Lu T., Qiu T., Han B., Wang Y., Sun X., Qin Y., Liu A., Ge N., Jiao W. (2021) *Circular RNA circCSNK1G3 induces HOXA10 signaling and promotes the growth and metastasis of lung adenocarcinoma cells through hsa-miR-143-3p sponging.* Cell Oncol. 44(2): 297–310. https://doi.org/10.1007/s13402-020-00565-x

Madhavan M., Ritchie A.J., Aboagye J., Jenkin D., Provstgaad-Morys S., Tarbet I., Woods D., Davies S., Baker M., Platt A. et al. (2022) *Tolerability and immunogenicity of an intranasally-administered adenovirus-vectored COVID-19 vaccine: An open-label partially-randomised ascending dose phase I trial.* eBioMedicine 85: 104298. https://doi.org/10.1016/j.ebiom.2022.104298

Maizel J.V., White D.O., Scharff M.D. (1968) *The polypeptides of adenovirus.* Virology 36(1): 115–125. https://doi.org/10.1016/0042-6822(68)90121-9

Porterfield J.Z., Zlotnick A. (2010) *A simple and general method for determining the protein and nucleic acid content of viruses by UV absorbance.* Virology 407(2): 281–288. https://doi.org/10.1016/j.virol.2010.08.015
